# A Novel Multivariate Analysis: Overturning Long‐Held Beliefs About Non‐Photochemical Quenching

**DOI:** 10.1111/ppl.70420

**Published:** 2025-07-19

**Authors:** Lennart A. I. Ramakers, Jeremy Harbinson, Herbert van Amerongen

**Affiliations:** ^1^ Laboratory of Biophysics Wageningen University Wageningen the Netherlands

**Keywords:** dark‐ and light‐adapted leaves, multivariate analysis, non‐photochemical quenching, PsbS, zeaxanthin

## Abstract

When light absorption exceeds photochemical quenching, plants activate non‐photochemical quenching (NPQ) to dissipate excess energy as heat. Recently, we have developed a novel multivariate pipeline for NPQ induction analysis. Applying this pipeline to NPQ induction curves of several *Arabidopsis thaliana* NPQ genotypes, overturns the long‐held belief that zeaxanthin (Zx) accelerates NPQ induction upon light‐adaptation. We demonstrate that the observed acceleration is solely due to the action of PsbS. Our approach allows the synergistic inter‐relationships between PsbS and Zx to be unambiguously explored. Specifically, we applied our analysis to dark‐ and light‐adapted wild‐type (wt), Zx‐lacking (*npq1*), Zx‐rich (*npq2*) and PsbS‐lacking (*npq4*) *A. thaliana* . Only the PsbS‐dependent quenching in *npq2*, wt and *npq1* plants exhibited faster induction kinetics following light adaptation. Changes in the Zx‐levels (*npq1* → wt → *npq2*) lead to changes in the overall amplitudes of the PsbS‐components, revealing a Zx‐driven amplification of PsbS‐dependent quenching. In the presence of PsbS (*npq2*/wt), Zx also provides its own distinct contribution to NPQ. Together, this reveals the distinct roles of Zx in NPQ and the multilayered synergistic relationship between PsbS and Zx. Combined with mutant genotypes, our unique analysis is an invaluable toolkit to answer mechanistic questions and will allow different NPQ models to be experimentally explored.

## 1 Introduction

Photosynthesis is the process by which plants convert light energy, available in solar radiation, to chemical energy, which can be used and stored by plants and animals (Nelson and Yocum 2006; Blankenship 2008; Johnson 2016). Broadly speaking, this process can be separated into two parts. The first is called the ‘light’ reactions, while the second is collectively called the ‘dark’ reactions. The ‘light’ reactions are responsible for capturing incident photons and converting light energy into metabolically useful chemical energy. The ‘light’ reactions are underpinned by the photosystem I (PSI) and II (PSII) supercomplexes, which contain photosynthetic pigments and capture the incoming photons. The light energy absorbed by PSI and PSII is used to drive linear electron transport from water to NADPH and to accumulate protons in the lumenal space. The accumulation of protons in the thylakoid lumen establishes a proton ‘gradient’ across the thylakoid membrane, which is used by the ATP‐synthase to generate ATP (Nelson and Yocum 2006; Johnson 2016). The dark reactions then use ATP and NADPH in the Calvin‐Benson‐Bassham (CBB) cycle to fix atmospheric CO_2_ into sugars and starch. Initially, as the light intensity increases, the rate at which the ‘light’ reactions generate ATP and NADPH increases, increasing the rate at which the CBB cycle can fix CO_2_. In parallel with the ‘light’ reactions and the CBB cycle, the acidification of the lumenal space also leads to the activation of several mechanisms underpinning non‐photochemical quenching (NPQ) (Demmig‐Adams and Adams 1996b; Horton et al. 1996; De Bianchi et al. 2010; Ruban et al. 2012). As the light intensity continues to increase, the overall amount of NPQ present also increases, and there is a point at which the ‘light’ reactions and the CBB cycle reach their maximum rates. Above this intensity, the rate of photon absorption is greater than the capacity of photochemistry of PSI and PSII, which leads to the progressive saturation of the photosynthetic electron transport or CO_2_ assimilation and the further buildup of NPQ with increasing irradiance. The result of these responses is that the accumulated NPQ minimises the production of chlorophyll triplet states, which can easily form when excitations encounter closed PSII RCs (Vass 2011; Telfer 2014). These triplets can lead to the production of highly reactive singlet oxygen, resulting in photodamage of the photosynthetic apparatus (Demmig‐Adams and Adams 1996b; Horton et al. 1996; De Bianchi et al. 2010; Ruban et al. 2012). In PSII, the fastest of these mechanisms are underpinned by the protonation of the PsbS protein and the accumulation of zeaxanthin (Zx) via the activation of the violaxanthin de‐epoxidase (VDE) enzyme (Demmig‐Adams and Adams 1996a, 1996b; D'Haese et al. 2004; Li et al. 2004, 2009; Johnson et al. 2009; Jahns and Holzwarth 2012; Ruban et al. 2012; Sylak‐Glassman et al. 2014; Goldschmidt‐Clermont and Bassi 2015; Armbruster et al. 2016; Ruban 2016, 2019; Farooq et al. 2018; Townsend et al. 2018; Van Amerongen and Chmeliov 2020; Ruban and Wilson 2021; Long et al. 2022).

One of the most common techniques used to measure and explore NPQ is via fluorescence spectroscopy, such as pulse‐amplitude‐modulated (PAM) fluorimetry (Maxwell and Johnson 2000; Baker 2008; Harbinson 2018). Commonly, the Stern‐Volmer equation ($\mathrm{NPQ}=\left(F_{m}-F_{m}^{\prime }\right)/F_{m}^{\prime }$) is used to determine an NPQ value every time a saturating pulse is used to close the PSII RCs and produce the $F_{m}$ (or $F_{m}^{\prime }$) fluorescence intensity (where $F_{m}$ and $F_{m}^{\prime }$ are the fluorescence intensity obtained when all PSII RCs are closed without and with NPQ, respectively) (Butler 1978; Bilger and Björkman 1990; Maxwell and Johnson 2000; Harbinson 2018). Due to the ease with which NPQ can be determined with this method, it has been widely applied to probe changes in the overall size and induction profile of NPQ in wild‐type (wt) and genetically altered plants (Li et al. 2000, 2009; Johnson and Ruban 2010; Sylak‐Glassman et al. 2014; Armbruster et al. 2016). This method can also probe changes in NPQ size and induction caused by different abiotic stresses or conditions. One particularly important abiotic condition is the initial state of photosynthesis prior to NPQ induction. Basically, the photosynthetic machinery can either be in the dark‐adapted state or a light‐adapted state prior to NPQ induction. In the dark‐adapted state, both the ‘light’ reactions and the CBB cycle are idle, almost all of the PSII RCs are in their open state and there is no Zx or antheraxanthin present (Kromdijk et al. 2016). This state is present before dawn, after leaves have spent the night in darkness. Contrasting this, the light‐adapted state of the photosynthetic apparatus develops during illumination. In this state, both the ‘light’ reactions and the CBB cycle have been or are still active, not all RCs are necessarily open and most likely both antheraxanthin and Zx are present at measurable levels (Kromdijk et al. 2016). This state is thought to be present throughout the day (Long et al. 2022; Schiphorst et al. 2023). Together, the dark‐ and light‐adapted states are representative throughout the day and therefore, to fully understand the NPQ induction, both states must be explored (Johnson et al. 2008; Steen et al. 2020; Long et al. 2022). One of the more notable differences between the states is the significantly faster NPQ induction in light‐adapted leaves. Several studies have ascribed these faster kinetics to either the presence of Zx in light‐adapted leaves or to a complicated combination of actions by both PsbS and Zx (Johnson et al. 2008; Steen et al. 2020). Faster kinetics have also recently been seen in frequency‐domain chlorophyll fluorescence measurements, which employ an oscillating actinic light source and empirically analyse the oscillating fluorescence response of the plants (Nedbal and Lazár 2021; Niu et al. 2023, 2024). These measurements were performed on light‐adapted leaves and found a fast ‘resonance’ response with a period of 30 s in several *Arabidopsis thaliana* plants, including the wt and *npq1* (Zx‐lacking). This fast ‘resonance’ was absent in the fluorescence response of *npq4* (PsbS‐lacking) *A. thaliana* plants, suggesting that PsbS played a role in these fast responses (Nedbal and Březina 2002; Nedbal and Lazár 2021; Lazár et al. 2022; Niu et al. 2023, 2024).

Here we apply the multivariate analysis pipeline we recently developed (Ramakers et al. 2025) to datasets of NPQ induction curves obtained from dark‐ and light‐adapted leaves. Using PAM fluorimetry with red actinic light (Figure S1b), dark‐ and light‐adapted datasets were obtained for wt, Zx‐lacking (*npq1*), Zx‐rich (*npq2*) and PsbS‐lacking (*npq4*) *A. thaliana* plants. The analysis reveals that NPQ induction in both the dark‐ and light‐adapted leaves is underpinned by five distinct components. These components contribute in a unique way to each of the plant genotypes studied, providing insights into the role(s) of Zx and PsbS in both dark‐ and light‐adapted NPQ induction.

## 2 Materials and Methods

### 2.1 Materials

All *A. thaliana* ((L.) Heynh.) plants were grown in a Hettich ESP PRC 1200 WL growth cabinet (Hettich; https://www.hettichbenelux.com). Plants were grown in short day conditions (8/16 h light/dark) at an actinic light intensity of 125 μmol m^−2^ s^−1^ (spectrum shown in Figure S1a), daytime temperature of 24°C, night time temperature of 22°C and relative humidity of 60%. Seeds of wt, *npq1, npq2* and *npq4* *A. thaliana* (Columbia‐0) were sown on soil and allowed to germinate and grow for 6–7 weeks. All leaves were measured at the same developmental stage, obtained from 6 to 7‐week‐old plants.

Nigericin, 3‐(3,4‐Dichlorophenyl)‐1,1‐dimethylurea (DCMU) and ethanol were purchased from Sigma‐Aldrich and D,L‐dithiothreitol (DTT) was purchased from Fluka chemicals; all chemicals were used without further purification. 5 mM stock solutions were prepared for nigericin and DCMU using ethanol and a 50 mM aqueous stock solution was prepared for DTT. These stock solutions were used to prepare the 5 mM DTT, 5 mM DTT and 50 μM nigericin and 50 μM DCMU aqueous solutions. Leaves were chemically infiltrated by being immersed in these solutions for ~2 h prior to the chemical‐treatment measurements.

### 2.2 Pulse Amplitude Modulated (PAM) Fluorometry

Experiments were carried out using a Walz mini‐PAM Fluorimeter (Walz; https://www.walz.com). Briefly, in these measurements, *A. thaliana* leaves, dark‐adapted overnight, were detached from the plant just prior to the measurement and placed into a small amount of water in a Petri dish. Using a multi‐core optical fibre, the measuring light and saturating pulses (0.8 s duration and an intensity of 7000 μmol m^−2^ s^−1^) of the mini‐PAM were directed midway along the leaf on the adaxial side, avoiding the mid‐vein. Chlorophyll fluorescence emitted from the adaxial leaf surface was collected by the same multi‐core optical fibre. The actinic light scheme and saturating pulse sequence were generated and controlled by a batch file in the WinControl‐3.30 Walz PAM software. The actinic light scheme consisted of 25 s of darkness, followed by two cycles of 10 min of illumination, followed by 5 min of post‐illumination dark recovery. These measurements were used to obtain the dark‐ (first cycle) and light‐adapted (second cycle) NPQ induction datasets. For the saturating pulses, the following sequence was used: An initial saturating pulse was utilised to measure the dark‐adapted state at the start of the experiment, this pulse was followed by a series of pulses spaced at 10 s intervals for the first 2 min of illumination (ensuring sufficient sampling of the faster kinetic components). Finally, a series of saturating pulses, spaced at 30 s intervals, was used for the rest of the experiment. Whilst this high‐resolution pulse sequence was previously shown (Ramakers et al. 2025) to be slightly actinic in nature, it did not affect the profiles of the extracted NPQ components. Additional light response curve measurements consisting of 7 illumination steps, lasting 10 min each, were performed for wt *A. thaliana*. Red actinic light intensities of 25, 64, 123, 281, 621, 811 and 1135 μmol m^−2^ s^−1^ were utilised for the steps in these light response curve measurements. Between each of these illumination steps, the actinic light was turned off for 10 s, to allow $F_{o}^{\prime }$ to be determined for each illumination intensity. These measurements were used to generate a wt light‐adapted NPQ induction dataset with a short post‐illumination dark recovery period (hereafter referred to as the light response curve dataset), allowing the effect of the 5 min of post‐illumination recovery on the light‐adaptation of the leaf to be explored. All measurements were repeated at least five times on separate leaves. The NPQ value was calculated using the Stern‐Volmer equation ($\mathrm{NPQ}=\left(F_{m}-F_{m}^{\prime }\right)/F_{m}^{\prime }$) and (1 − qP), a proxy for the fraction of closed RCs, was calculated using the expression $\left(1-\mathrm{qP}\right)=\left(F^{\prime }-F_{o}^{\prime }\right)/\left(F_{m}^{\prime }-F_{o}^{\prime }\right)$; (1 − qP)_ss_ is the value obtained at the end of the illumination period, when a steady‐state value has been reached. The measured NPQ induction curve dataset was then binned according to the calculated (1 − qP)_ss_ values prior to data analysis. All data analysis was performed utilising a custom‐made Python 3.9 analysis pipeline as previously described (Ramakers et al. 2025). Briefly, the analysis pipeline uses a combination of principal component analysis (Maćkiewicz and Ratajczak 1993; Fritzsch et al. 2018) and full‐harmonic phasor analysis (Bader et al. 2014; Franssen et al. 2020) to identify the distinct induction components underlying the NPQ dataset. Once identified, non‐negative matrix factorisation (Berry et al. 2007) is used to fully deconvolute the NPQ dataset into its underlying components (the analytical pipeline is summarised in Notes S1: Multivariate Analysis Pipeline Summary).

### 2.3 Ultrafast Fluorometry

Experiments were carried out using a streak camera (Hamamatsu, https://www.hamamatsu.com) with a Leukos Rock supercontinuum picosecond laser system (Leukos, https://www.leukos‐laser.com). The picosecond laser system produces 440 nm pulses (FWHM = 10 nm/pulse width ~10 ps) at a repetition rate of 38 MHz. These pulses were focused onto leaves in a rotating sample cell to excite fluorescence. The fluorescence was directed into an Andor Shamrock 500i (Oxford Instruments Group, https://andor.oxinst.com) spectrograph connected to the streak camera. For $F_{m}$ measurements, the sample cell rotated at 2 RPM, the 440 nm laser pulse intensity was 10 μW (spot size: ~100 μm). The sample was illuminated with an additional 1 mW 532 nm continuous wave (spot size: ~5 mm) diode laser (Thorlabs, https://www.thorlabs.com) to ensure that all PSII reaction centres were closed. The measured streak spectra were analysed, allowing both the overall ($\tau_{F_{m}}$) and PSII only ($\tau_{F_{m}}^{\text{PSII}}$) average lifetimes in the $F_{m}$ state to be obtained for wt, *npq1*, *npq2* and *npq4* plants. For wt and *npq2*, these measurements were repeated with DCMU‐infiltrated leaves. All measurements were repeated three times. These measurements were used to determine if any of the dark‐adapted mutant genotypes (*npq1*, *npq2* and *npq4*) exhibited long‐term quenching. If any of the mutant genotypes exhibit long‐term quenching, the overall lifetimes were used to correct the PAM data for this quenching ($F_{m}^{\mathrm{No}\;\mathrm{NPQ}}=F_{m}^{\text{Observed}}\left(\tau_{F_{m}}^{\mathrm{No}\;\mathrm{NPQ}}/\tau_{F_{m}}^{\text{Observed}}\right)$, outlined in Notes S2: Long‐term $F_{m}$ quenching Correction and Figure S3).

## 3 Results

### 3.1 Dark‐ and Light‐Adapted NPQ Induction in wt *A. thaliana*

**FIGURE 1:**
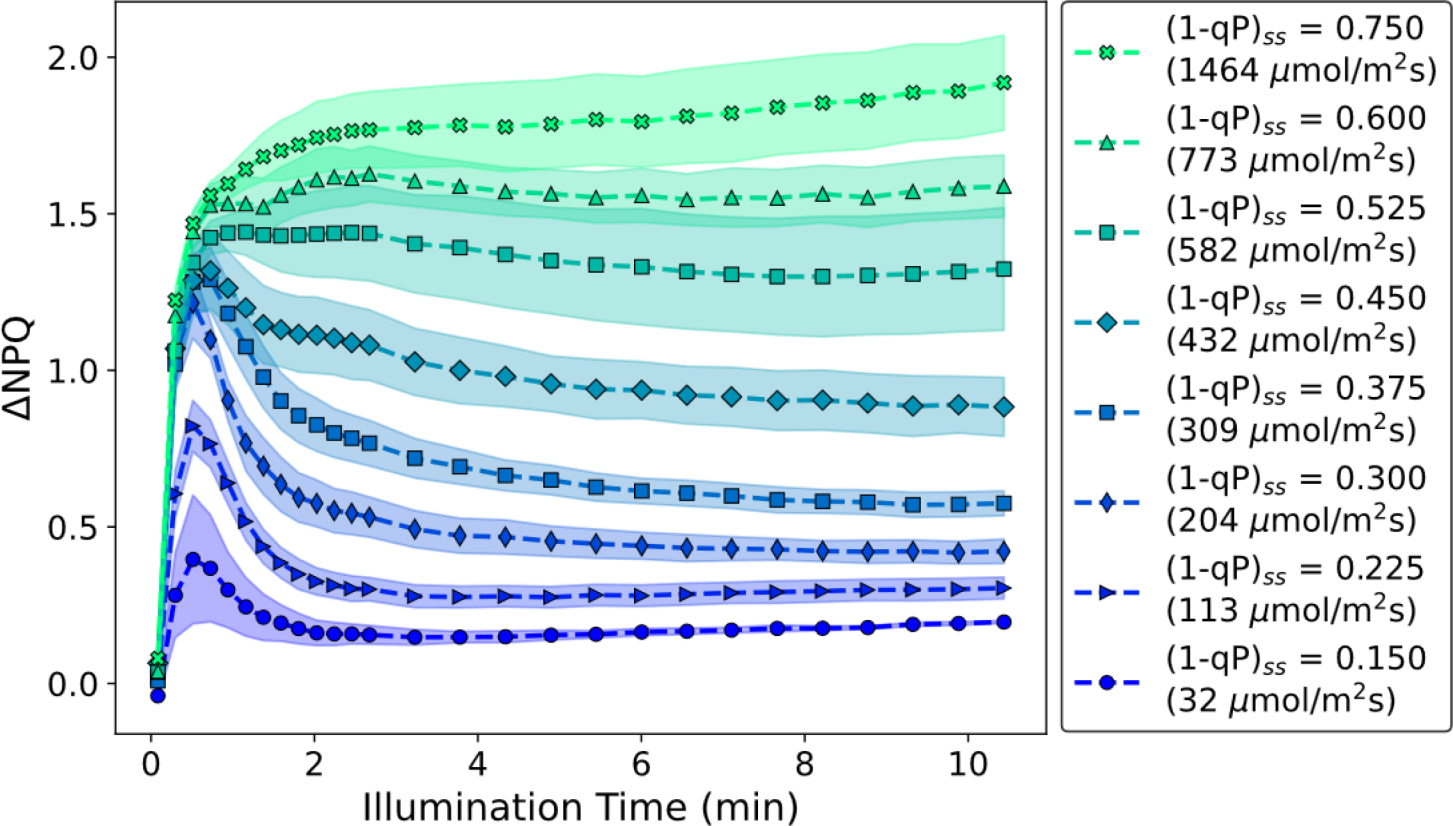
NPQ induction curves obtained for light‐adapted wt *A. thaliana* plants at a range of different actinic light intensities, leading to differing values of (1 − qP)_ss_. Each curve is a binned average of individual measurements carried out on separate leaves; the shaded area shows the associated standard error.

In the case of fully dark‐adapted plants (Ramakers et al. 2025), the NPQ induction curves approach an almost stable steady‐state level after approximately 4 min of illumination. As previously described, as the light intensity is increased, leading to higher values of (1 − qP)_ss_, the induction curve profile undergoes non‐trivial changes (Ramakers et al. 2025). These cannot be explained by a simple linear combination of low and high (1 − qP)_ss_ NPQ induction curves (Ramakers et al. 2025). At low (1 − qP)_ss_ values (≤ 0.275), these curves show a distinct local NPQ maximum within the first 2 min of illumination, followed by a gradual decrease to a lower steady‐state NPQ value (Ramakers et al. 2025). As (1 − qP)_ss_ increases, this local maximum occurs at longer illumination times, shifting from ~1 to ~2 min. When the (1 − qP)_ss_ values are > 0.3, the NPQ induction profile becomes monotonically increasing (Ramakers et al. 2025). In Figure 1 the NPQ induction curves are shown for wt *A. thaliana* plants that have been allowed to undergo light adaptation. Light‐ and dark‐adapted (Ramakers et al. 2025) plants yield distinct induction kinetics and profiles, with light‐adapted plants exhibiting noticeably faster kinetics. Average NPQ induction curves are shown as a function of (1 − qP)_ss_ (a proxy for the fraction of closed PSII RCs) instead of the commonly used light intensity.

When the plants start in a light‐adapted state (meaning there is already some NPQ present), the induction of additional NPQ (ΔNPQ) leads to profiles which hint at faster induction kinetics. Specifically, the ΔNPQ induction curves reach a nearly steady‐state ΔNPQ value after only 1–2 min of illumination (Figure 1). These accelerated light‐adapted kinetics are also seen in the light response curve dataset (Figure S4). Again, as (1 − qP)_ss_ increases, these induction curves exhibit a number of intricate changes. At low values of (1 − qP)_ss_ these ΔNPQ induction profiles contain a local maximum after ~30 s of illumination, before decreasing to a lower value. As (1 − qP)_ss_ increases, the profile of the induction curve changes until it becomes a monotonically increasing curve (when (1 − qP)_ss_ > 0.525), reaching a steady‐state value of ΔNPQ after only ~1 min of illumination. As for the dark‐adapted dataset (Ramakers et al. 2025), a simple linear‐combination model fails to accurately describe the ΔNPQ induction curves at intermediate values of (1 − qP)_ss_ (Figure S5; (1 − qP)_ss_ ≈ 0.3 → 0.75).

**FIGURE 2:**
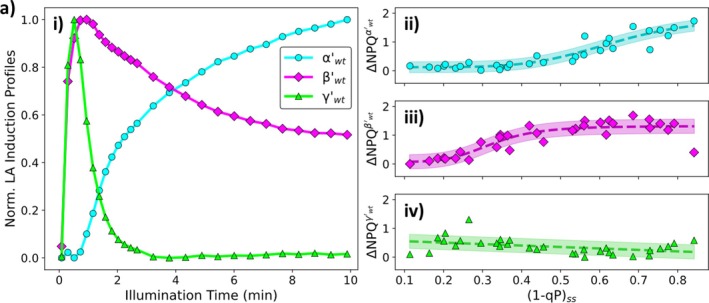
(a) (i) Normalised ΔNPQ components for light‐adapted wt *A. thaliana* and their contribution to the overall ΔNPQ, (ii) $\alpha \prime_{\mathrm{wt}}$ (sigmoidal with a turning point at (1 − qP)_ss_ = (0.64 ± 0.08) and a maximum of (1.8 ± 0.6)), (iii) $\beta \prime_{\mathrm{wt}}$ (sigmoidal with a turning point at (1 − qP)_ss_ = (0.32 ± 0.03) and a maximum of (1.2 ± 0.2)) and (iv) $\gamma \prime_{\mathrm{wt}}$ (linear with a gradient of (−0.5 ± 0.2) and an intercept of (0.6 ± 0.1)), respectively. All plants were illuminated using red actinic light.

To further explore these changes, a dark‐adapted (consisting of 85 individual measurements performed on randomly selected leaves) and a light‐adapted series (consisting of 32 individual measurements performed on randomly selected leaves) of wt *A. thaliana* NPQ induction datasets were analysed with our multivariate pipeline. This pipeline, developed using NPQ induction datasets for wt and *npq1 A. thaliana* (Ramakers et al. 2025), identifies and deconvolutes an NPQ induction dataset into a number of kinetically distinct NPQ components and estimates the amplitudes of these components as a function of (1 − qP)_ss_. For the dark‐adapted wt plants, the pipeline reveals that the dataset is underpinned by three kinetically distinct components, as reported previously (Ramakers et al. 2025). Briefly, the slowest component was shown to mainly arise from the accumulation of Zx (also referred to as qZ or as part of qE), whereas the other two components are thought to be associated with the protonation of the PsbS protein (also referred to as qE) upon illumination. The results of this analysis for the light‐adapted wt *A. thaliana* NPQ induction dataset are summarised in Figure 2.

For light‐adapted plants the analysis pipeline reveals that the induction curves can also be described by three components (Figure 2a). The slowest component ($\alpha \prime_{\mathrm{wt}}$) follows a simple monotonically increasing profile over illumination time (cyan circles, Figure 2a(i)) and has a very similar shape to the dark‐adapted $\alpha_{\mathrm{wt}}$ component, including the distinct short lag at the start of the illumination period (Ramakers et al. 2025). This similarity indicates that the $\alpha \prime_{\mathrm{wt}}$ component arises from the further accumulation of Zx under light‐adapted conditions (qZ/part of qE). Its contribution to the overall ΔNPQ follows a sigmoidal curve with increasing (1 − qP)_ss_ at the end of the second illumination cycle (Figure 2a(ii)). Comparing the turning points of the contributions of the $\alpha_{\mathrm{wt}}$ (dark‐adapted) and $\alpha \prime_{\mathrm{wt}}$ (light‐adapted) components to the overall ΔNPQ reveal that light‐adaptation increases the (1 − qP)_ss_ value at which this inflection point occurs by ~1.7‐fold. This increase is consistent with the presence of Zx in the light‐adapted state and the residual NPQ at the start of the second induction cycle is apparently due to the previously accumulated Zx. The other two faster induction components ($\beta \prime_{\mathrm{wt}}$ and $\gamma \prime_{\mathrm{wt}}$) exhibit more complicated kinetics, with both containing a local ΔNPQ maximum before decreasing to a lower steady‐state value of ΔNPQ (magenta diamonds and lime green triangles, respectively; Figure 2a(i)). For the $\beta \prime_{\mathrm{wt}}$ component, this local ΔNPQ maximum occurs after approximately 45 s of illumination. Following this local maximum the $\beta \prime_{\mathrm{wt}}$ component then decreases to a lower steady‐state value (magenta diamonds, Figure 2a(i)). The contribution of this component to the overall ΔNPQ (Figure 2a(iii)) follows a sigmoidal curve as (1 − qP)_ss_ increases. Overall, these aspects show that the $\beta \prime_{\mathrm{wt}}$ component is the light‐adapted counterpart of $\beta_{\mathrm{wt}}$ (qE) (Ramakers et al. 2025). The $\gamma \prime_{\mathrm{wt}}$ component is the fastest induction component found for the light‐adapted wt plants. Its induction profile reaches a local maximum after ~25 s of illumination. Following this local maximum, this component rapidly decreases, almost vanishing completely after about 3 min of illumination (lime green triangles, Figure 2a(i)). Furthermore, this component provides an almost constant contribution to the overall ΔNPQ (Figure 2a(iv)). Combined with the fast induction kinetics and the linear trend with (1 − qP)_ss_, this component (lime green triangles, Figure 2a) is thought to be the light‐adapted version of the $\gamma_{\mathrm{wt}}$ component (qE) in dark‐adapted plants (Ramakers et al. 2025). Both the $\gamma_{\mathrm{wt}}$ and $\gamma \prime_{\mathrm{wt}}$ components are thought to be consistent with the combined actions of PsbS and the K^+^‐efflux antiporter (Armbruster et al. 2014, 2016), as is also confirmed by measurements on a mutant lacking this component (unpublished results). Finally, the light response curve dataset can also be deconvoluted using the light‐adapted ($\alpha \prime_{\mathrm{wt}}$, $\beta \prime_{\mathrm{wt}}$ and $\gamma \prime_{\mathrm{wt}}$) NPQ components (Figure S6) revealing that reducing the post‐illumination recovery time from 5 min to 10 s has no effect on the underlying light‐adapted components. This indicates that the 5 min recovery period does not significantly disturb the light‐adapted state of the leaf.

### 3.2 Dark‐ and Light‐Adapted NPQ Induction in *npq2* *A. thaliana*

The presence of Zx has been concluded to be solely responsible for the faster NPQ induction kinetics in light‐adapted leaves, chloroplasts and isolated light‐harvesting complexes (Ruban and Horton 1999; Johnson et al. 2008). To further explore this observation and the effect of Zx on the induction of NPQ in light‐adapted leaves, NPQ induction datasets for both dark‐ and light‐adapted *npq2 A. thaliana* plants were measured. These mutant plants lack an active zeaxanthin epoxidase enzyme and are always Zx‐rich. The mutation causes a small amount (${\mathrm{NPQ}}_{\tau }^{\text{long}-\text{term}}$ = (0.28 ± 0.03)) of long‐term $F_{m}$ quenching (Figure S3) and profile changes in the NPQ induction curves. Using the overall lifetimes obtained for wt and *npq2* DCMU (a linear electron transport inhibitor which permanently closes all PSII RCs) (Hsu et al. 1986) treated leaves, a correction factor was calculated to obtain a $F_{m}$ value for each of the *npq2* PAM measurements ($F_{m}^{\mathrm{No}\;\mathrm{NPQ}}=F_{m}^{\text{Observed}}\left(\tau_{F_{m}}^{\mathrm{No}\;\mathrm{NPQ}}/\tau_{F_{m}}^{\text{Observed}}\right)$, outlined in Notes S2: Long‐term $F_{m}$ quenching Correction and Figure S3). Following this correction, the NPQ induction curves show that dark‐ and light‐adapted *npq2* leaves reach similar levels of NPQ as wt plants (Figure S7). Broadly speaking, the dark‐ and light‐adapted NPQ induction curves for *npq2* share several features with the wt curves (Figure 1, Figure S8). Specifically, both datasets show NPQ induction curves which contain a local maximum after 1–2 min of illumination in dark‐adapted leaves and after ~30 s in light‐adapted leaves. As (1 − qP)_ss_ increases, the profile of the induction curves transitions to a simple monotonically increasing curve, again echoing the changes seen in the wt NPQ induction datasets. However, the permanent presence of Zx leads to a distinct increase in the amplitudes of the local maxima and in the dark‐adapted case, the NPQ induction curves show an increase in the onset kinetics in *npq2*. To further explore these change(s) the analysis pipeline was used to extract the components underpinning the *npq2* induction datasets.

**FIGURE 3:**
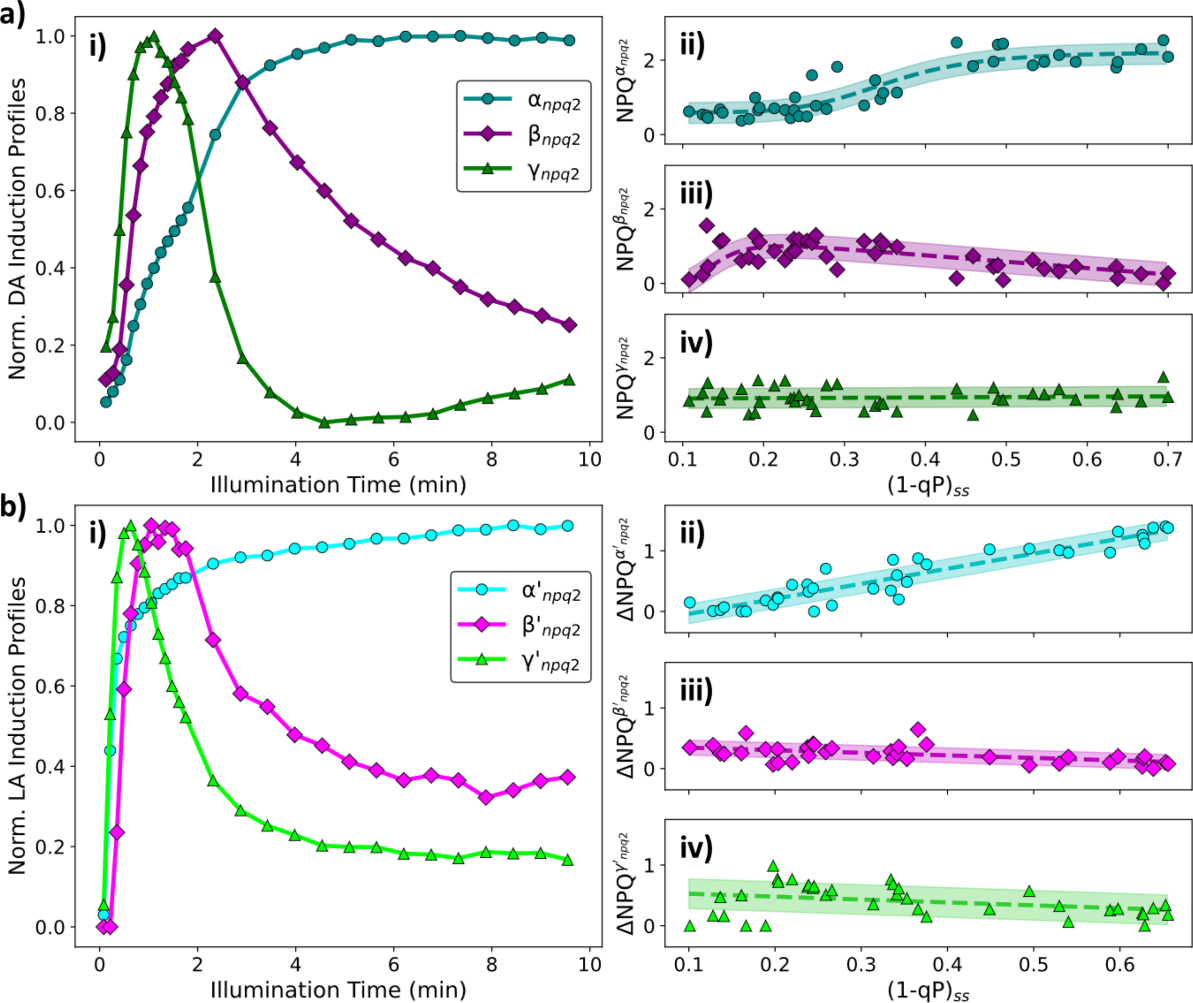
(a) (i) Normalised NPQ components for dark‐adapted *npq2* *A. thaliana* and their contribution to the overall NPQ, (ii) $\alpha_{\mathrm{npq}2}$ (sigmoidal with a turning point at (1 − qP)_ss_ = (0.40 ± 0.09) and a maximum of (2.3 ± 0.3)), (iii) $\beta_{\mathrm{npq}2}$ (sloped sigmoidal with a turning point at (1 − qP)_ss_ = (0.15 ± 0.03) and a maximum of (1.1 ± 0.1)) and (iv) $\gamma_{\mathrm{npq}2}$ (linear with a gradient of (−0.2 ± 0.2) and an intercept of (0.9 ± 0.1)), respectively. (b) (i) Normalised ΔNPQ components for light‐adapted *npq2* *A. thaliana* and their contribution to the overall ΔNPQ, (ii) $\alpha \prime_{\mathrm{npq}2}$ (linear with a gradient of (2.5 ± 0.2) and an intercept of (−0.3 ± 0.1)), (iii) $\beta \prime_{\mathrm{npq}2}$ (linear with a gradient of (−0.3 ± 0.1) and an intercept of (0.35 ± 0.04)) and (iv) $\gamma \prime_{\mathrm{npq}2}$ (linear with a gradient of (−0.4 ± 0.4) and an intercept of (0.6 ± 0.1)), respectively. All plants were illuminated using red actinic light.

Both the dark‐ and light‐adapted datasets appear to be underpinned by three distinct induction components. For dark‐adapted leaves, the slowest one (cyan circles, Figure 3a(i)) is a monotonically rising curve, approaching an asymptote after ~5 min. The contribution of this curve to the overall NPQ was sigmoidal with increasing (1 − qP)_ss_ (Figure 3a(ii)). Taken together, this component shares several features with the slowest dark‐adapted wt induction component (Ramakers et al. 2025), indicating that these components arise from the same molecular processes. The faster components (magenta diamonds and green triangles, Figure 3a(i)) both exhibit a local NPQ maximum within the first 2 min of illumination. Following this maximum, both of these components show a decrease in NPQ with the slower of these two components (magenta diamonds, Figure 3a(i)) approaching a lower steady‐state value. The contribution of this component follows a sloped sigmoid curve with increasing (1 − qP)_ss_, with a maximum contribution at (1 − qP)_ss_ ≈ 0.22 (Figure 3a(iii)). For the fastest component this decrease is followed by a gradual increase towards the end of the illumination period (green triangles, Figure 3a(i)). This component provides an almost constant contribution over the entire (1 − qP)_ss_ range (green triangles, Figure 3a(iv)). Again, these components are consistent with the two fastest components underpinning the dark‐adapted wt induction dataset (Ramakers et al. 2025). Whilst these fastest components are very similar to their wt counterparts, overall they exhibit higher amplitudes in *npq2* (~11% higher for β and ~80% higher for γ).

Two of the components underlying the ΔNPQ induction in light‐adapted *npq2* leaves also exhibit distinct local maxima before decreasing to a lower steady‐state value. The maxima of these two components occurs after ~35 s (green triangles, Figure 3b(i)) and ~1 min (magenta diamonds, Figure 3b(i)) of illumination. The contributions of these components vary linearly with (1 − qP)_ss_ but exhibit only a small gradient and therefore are approximately constant (Figure 3b(iii,iv)). The third component, which underpins the light‐adapted NPQ induction curves, follows a rapidly increasing monotonic profile and approaches an asymptote over the illumination period (cyan circles, Figure 3b(i)). The contribution of this component to the overall ΔNPQ also follows a linear trend with (1 − qP)_ss_ (cyan circles, Figure 3b(ii)).

To probe the processes which underpin the components revealed by the analysis pipeline, chemical treatments were performed. Since Zx is always present in *npq2* plants it is not possible to selectively chemically eliminate the effect of Zx accumulation with the use of DTT (Neubauer 1993) and so these plants were only treated with either nigericin, a pH decoupling agent (Horton et al. 2000; Brooks et al. 2013), or DCMU (Hsu et al. 1986). Under low light conditions ((1 − qP)_ss_ ≈ 0.15) nigericin‐induced difference in the NPQ and ΔNPQ induction curves (Figure S9) have very similar profiles to the β and γ components (Figure 3), indicating these arise from the protonation of PsbS (qE). DCMU treatments led to a significant loss of NPQ, resulting in slow monotonically increasing induction curves (Figure S10). This does not match any of the pipeline identified components underpinning the NPQ inductions in untreated *npq2*. Together with the nigericin treatment, this shows that the α components most likely arise from Zx‐dependent quenching (qZ/part of qE).

### 3.3 Dark‐ and Light‐Adapted NPQ Induction in *npq1* *A. thaliana*

**FIGURE 4:**
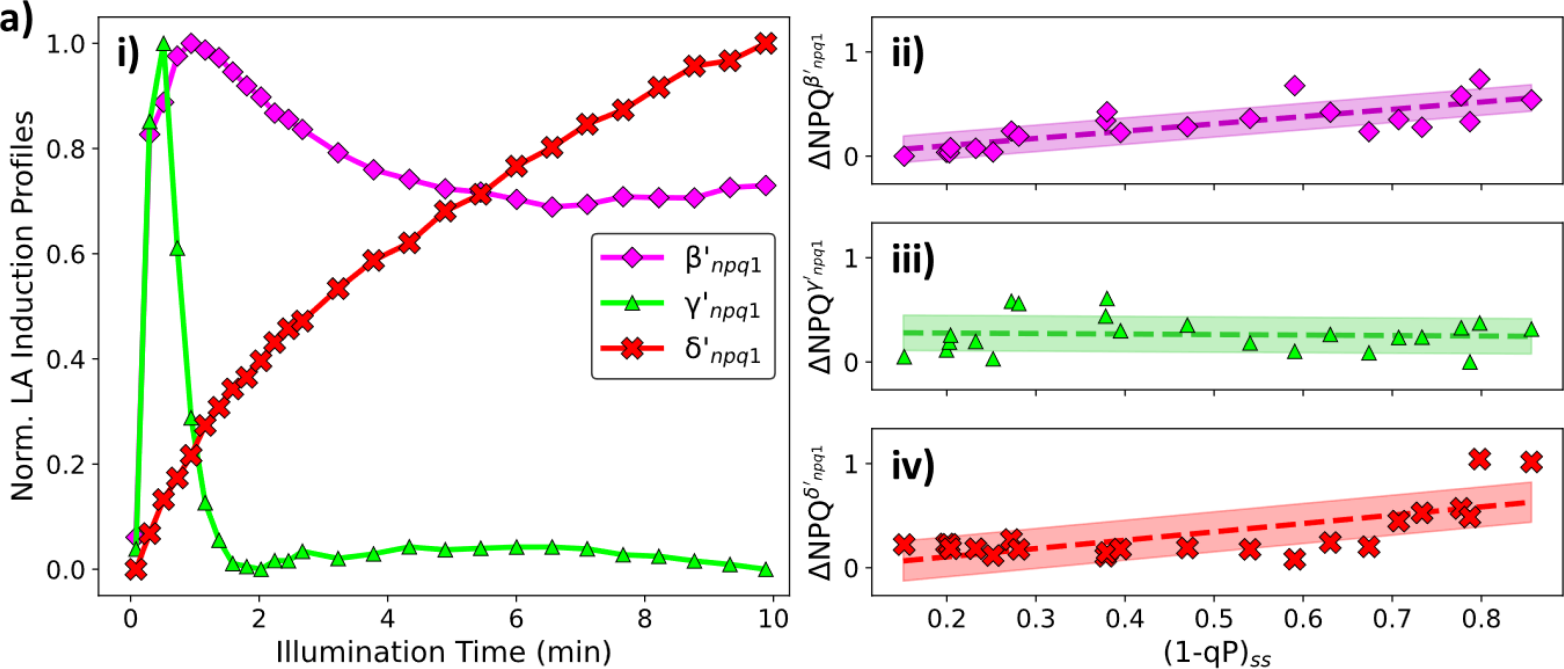
(a) (i) Normalised ΔNPQ components for light‐adapted *npq1* *A. thaliana* and their contribution to the overall ΔNPQ, (ii) $\beta \prime_{\mathrm{npq}1}$ (linear with a gradient of (0.7 ± 0.1) and an intercept of (−0.04 ± 0.07)), (iii) $\gamma \prime_{\mathrm{npq}1}$ (linear with a gradient of (−0.1 ± 0.2) and an intercept of (0.29 ± 0.09)) and (iv) $\delta \prime_{\mathrm{npq}1}$ (linear with a gradient of (0.8 ± 0.2) and an intercept of (−0.1 ± 0.1)), respectively.

To further explore the effect of Zx on the induction of NPQ in dark‐ and light‐adapted leaves, *npq1* plants were also measured. These plants contain a non‐functional version of the VDE enzyme and cannot accumulate Zx during illumination, though PsbS is present as usual. This lack of Zx accumulation leads to distinct changes in the profiles of the NPQ induction curves (Figure S11). In both dark‐ and light‐adapted leaves, the NPQ induction curves contain a distinct local maximum in the induced NPQ. For dark‐adapted leaves, this maximum occurs after ~1–2 min of illumination (Ramakers et al. 2025), whereas for light‐adapted leaves, this maximum in ΔNPQ occurs after ~25 s of illumination (Figure S11). In addition to the loss of the xanthophyll cycle leading to changes in the profile of the induction curves (Ramakers et al. 2025), the *npq1* mutation also leads to a marked decrease in the overall size of the induced NPQ (Figure 1, Figure S11). To reveal what effect(s) the loss of the presence of Zx has on NPQ induction, the analysis pipeline was used again. For the dark‐adapted plants, these three components have been described previously (Ramakers et al. 2025). Briefly, in the dark‐adapted plants, the loss of the xanthophyll cycle results in the ‘replacement’ of the component associated with the accumulation of Zx with a slower and lower‐amplitude $\delta_{\mathrm{npq}1}$ component, thought to arise from photo‐inhibitory (qI‐like) processes. Overall, the profiles of the faster $\beta_{\mathrm{npq}1}$ and $\gamma_{\mathrm{npq}1}$ are unaffected by the loss of the xanthophyll cycle, exhibiting similar profiles to their wt counterparts (qE) (Ramakers et al. 2025). The contributions of all these components to the overall NPQ are found to vary linearly with (1 − qP)_ss_. However, the contribution of the $\gamma_{\mathrm{npq}1}$ component to the overall NPQ varies the least, following a linear trend with only a small gradient consistent with the behaviour of its wt counterpart (Ramakers et al. 2025). The results of this analysis for the light‐adapted dataset are summarised in Figure 4.

As seen for the wt datasets, the pipeline reveals that the *npq1* light‐adapted dataset can also be described using three components. Following light‐adaptation the *npq1* ΔNPQ induction curves are also underpinned by three components (Figure 4a(i)). The slowest of these light‐adapted components ($\delta \prime_{\mathrm{npq}1}$; red crosses Figure 4a(i)) follows a simple monotonically increasing profile. The similarity of this profile to the $\delta_{\mathrm{npq}1}$ component (Figure S12a) underpinning the dark‐adapted dataset indicates that both profiles arise from the same underlying process, most likely photoinhibition (qI), which is unlikely to be significantly altered by light‐adaptation. The two faster components underlying the light‐adapted ΔNPQ induction curves ($\beta \prime_{\mathrm{npq}1}$ and $\gamma \prime_{\mathrm{npq}1}$, Figure 4a(i)) follow convoluted profiles containing local ΔNPQ maxima. For the $\beta \prime_{\mathrm{npq}1}$ component, this local maximum occurs after only 45 s of illumination and after this, the component undergoes a gradual decrease to a lower steady‐state value. The local maximum in the faster $\gamma \prime_{\mathrm{npq}1}$ component occurs after only 25 s before rapidly vanishing 85 s after the local peak in ΔNPQ. Interestingly, in the light‐adapted case, the profiles of the $\beta \prime_{\mathrm{npq}1}$ and $\gamma \prime_{\mathrm{npq}1}$ components are again very similar to their light‐adapted wt counterparts ($\beta \prime_{\mathrm{wt}}$ and $\gamma \prime_{\mathrm{wt}}$; Figure S13). The contribution of these three components to the overall induced ΔNPQ varies linearly with increasing (1 − qP)_ss_ (Figure 4a(ii,iii,iv)). The overall contributions of the $\beta \prime_{\mathrm{npq}1}$ and $\gamma \prime_{\mathrm{npq}1}$ (Figure 4a(ii,iii)) components are lower (~57% lower for β and ~25% lower for γ) than their light‐adapted wt counterparts (qE) ($\beta \prime_{\mathrm{wt}}$ and $\gamma \prime_{\mathrm{wt}}$, Figure 2a(iii,iv)). This is consistent with the dark‐adapted case where the contributions of both the $\beta_{\mathrm{npq}1}$ and $\gamma_{\mathrm{npq}1}$ components to the overall NPQ are significantly lower than their wt counterparts (~55% lower for β and ~60% lower for γ) (Ramakers et al. 2025).

### 3.4 Dark‐ and Light‐Adapted NPQ Induction in *npq4* *A. thaliana*

**FIGURE 5:**
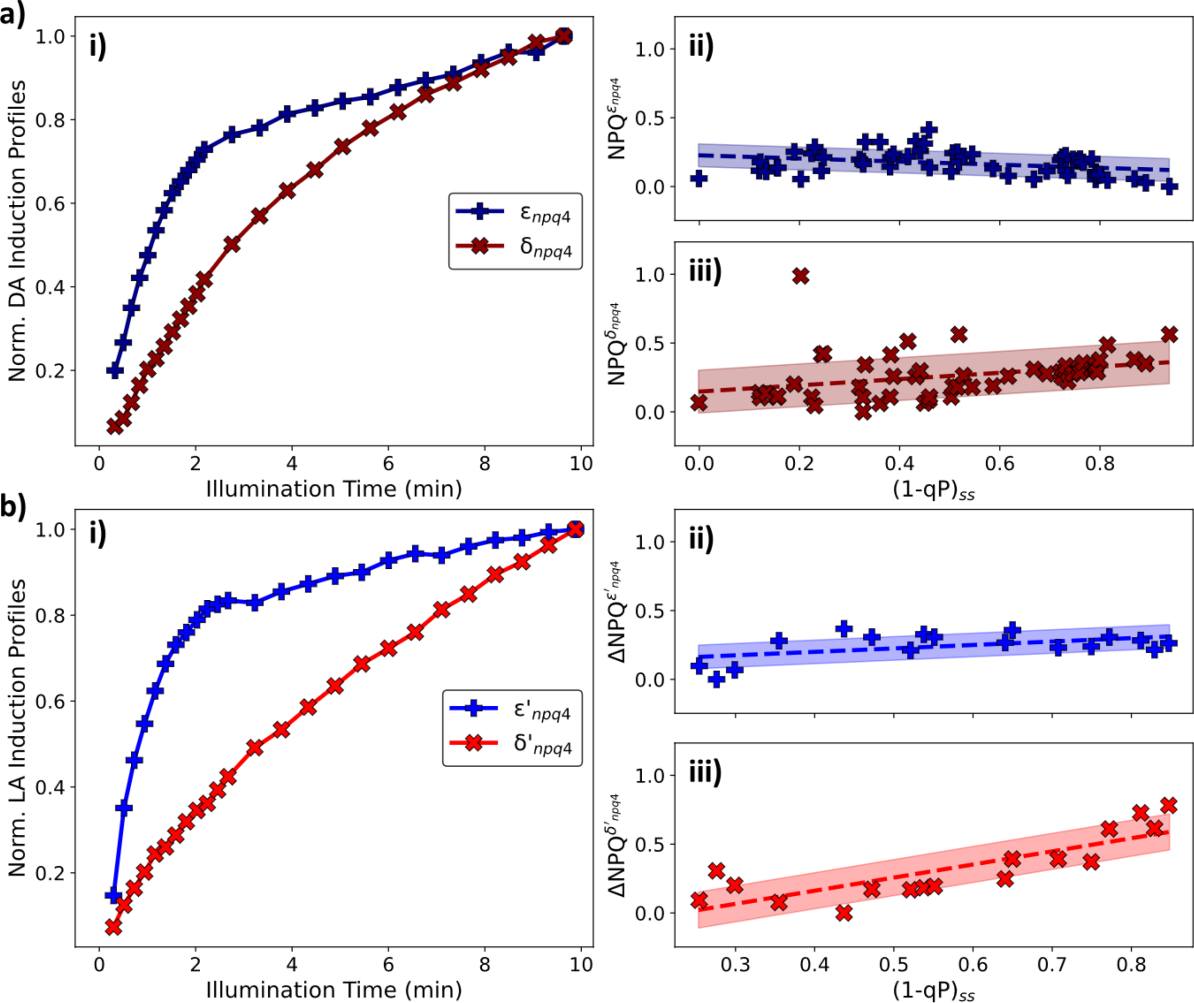
(a) (i) Normalised NPQ components for dark‐adapted *npq4* *A. thaliana* and their contribution to the overall NPQ, (ii) $\varepsilon_{\mathrm{npq}4}$ (linear with a gradient of (−0.11 ± 0.05) and an intercept of (0.23 ± 0.03)) and (iii) $\delta_{\mathrm{npq}4}$ (linear with a gradient of (0.2 ± 0.1) and an intercept of (0.15 ± 0.05)), respectively. (b) (i) Normalised NPQ components for light‐adapted *npq4* *A. thaliana* and their contribution to the overall ΔNPQ, (ii) $\varepsilon \prime_{\mathrm{npq}4}$ (linear with a gradient of (0.3 ± 0.1) and an intercept of (0.10 ± 0.07)) and (iii) $\delta \prime_{\mathrm{npq}4}$ (linear with a gradient of (1.0 ± 0.2) and an intercept of (−0.2 ± 0.1)), respectively. All plants were illuminated using red actinic light.

The effect of PsbS on the dark‐ and light‐adapted NPQ induction kinetics was explored using *npq4 A. thaliana* leaves. *npq4* plants completely lack the PsbS protein, and its removal leads to a marked decrease in both the induction rate and overall amount of NPQ induced upon illumination for dark‐ and light‐adapted leaves (Figure S14). Despite these marked changes, it was previously found that these plants exhibit both a small, rapidly reversible NPQ component (on the minute timescale) as well as a fully functional xanthophyll cycle (Horton et al. 2008; Johnson and Ruban 2010). The analysis pipeline was applied to explore the effect on the components underpinning the induction curves. The results are summarised in Figure 5.

The analysis pipeline reveals that both the dark‐ and light‐adapted *npq4* NPQ induction datasets are underpinned by two components with distinct induction kinetics (Figure 5a,b). For the dark‐adapted dataset both components follow monotonically increasing profiles. The faster of these two components ($\varepsilon_{\mathrm{npq}4}$; dark blue pluses in Figure 5a(i)) follows a more complicated induction profile, which increases more rapidly during the first 2 min of illumination before increasing more gradually for the rest of the illumination period, resulting in a distinct ‘kink’ feature appearing in its profile. Overall, this component only provides a small contribution to the overall NPQ and only varies weakly with increasing (1 − qP)_ss_ values following a linear trend with a small gradient (Figure 5a(ii)). Contrasting this the slower component ($\delta_{\mathrm{npq}4}$; dark red crosses in Figure 5a(i)) follows a simple monotonically increasing profile. The contribution of this component to the overall induced NPQ is seen to increase linearly with (1 − qP)_ss_, becoming the dominant component at high actinic light intensities (Figure 5a(iii)). Interestingly, the profiles of the two components ($\varepsilon \prime_{\mathrm{npq}4}$ and $\delta \prime_{\mathrm{npq}4}$; blue pluses and red crosses, respectively, in Figure 5b(i)) found to underpin the *npq4* light‐adapted ΔNPQ induction dataset are remarkably similar to their dark‐adapted counterparts ($\varepsilon_{\mathrm{npq}4}$ and $\delta_{\mathrm{npq}4}$; dark blue pluses and dark red crosses, respectively, in Figure 5a(i)). The close similarity demonstrates that in *npq4* the molecular mechanisms contributing to the induction of NPQ are unaffected by light‐adaptation (Figure S14). Furthermore, in the light‐adapted data the contributions of these components to the overall ΔNPQ vary linearly with increasing (1 − qP)_ss_ values (Figure 5b(ii,iii)).

Chemical treatments were utilised to explore the mechanisms underlying the NPQ induction components revealed by the analysis of the dark‐ and light‐adapted *npq4* datasets. DTT treatment (Neubauer 1993), which is known to inhibit the xanthophyll cycle, did not lead to significant changes in the profile of the induced NPQ in *npq4* leaves (Figure S15a). This shows that none of the identified components arises from Zx accumulation. Combining nigericin (Horton et al. 2000; Brooks et al. 2013) with DTT was found to change the NPQ induction in *npq4* leaves (Figure S15b,c). The combined DTT and nigericin treatment induced‐difference in the NPQ induction curve, which was found to exhibit a monotonically increasing profile containing a distinct ‘kink’ feature after about 2 min of illumination (Figure S15c). The shape of this treatment‐induced difference curve is very similar to the profile of the $\varepsilon_{\mathrm{npq}4}$ and $\varepsilon \prime_{\mathrm{npq}4}$ components (plus signs in Figure 5) showing that these components are caused by the acidification of the lumenal space (qE‐like). Both DCMU treatment, which permanently closes the PSII RCs, and the combined DTT & nigericin treatment, which inhibits the xanthophyll cycle and removes the pH difference across the thylakoid membrane, largely reduce the acidification of the lumen and so these treatments allow the approximate induction profile of qI‐like processes to be obtained. The similarity of the induction curves obtained after these treatments to the $\delta_{\mathrm{npq}4}$, $\delta_{\mathrm{npq}1}$, $\delta \prime_{\mathrm{npq}4}$ and $\delta \prime_{\mathrm{npq}1}$ components (crosses in Figures 4 and 5) demonstrate that these components arise from qI‐like processes (Figures S12 and S16).

## 4 Discussion

### 4.1 The Role(s) of Zx in NPQ Induction

**FIGURE 6:**
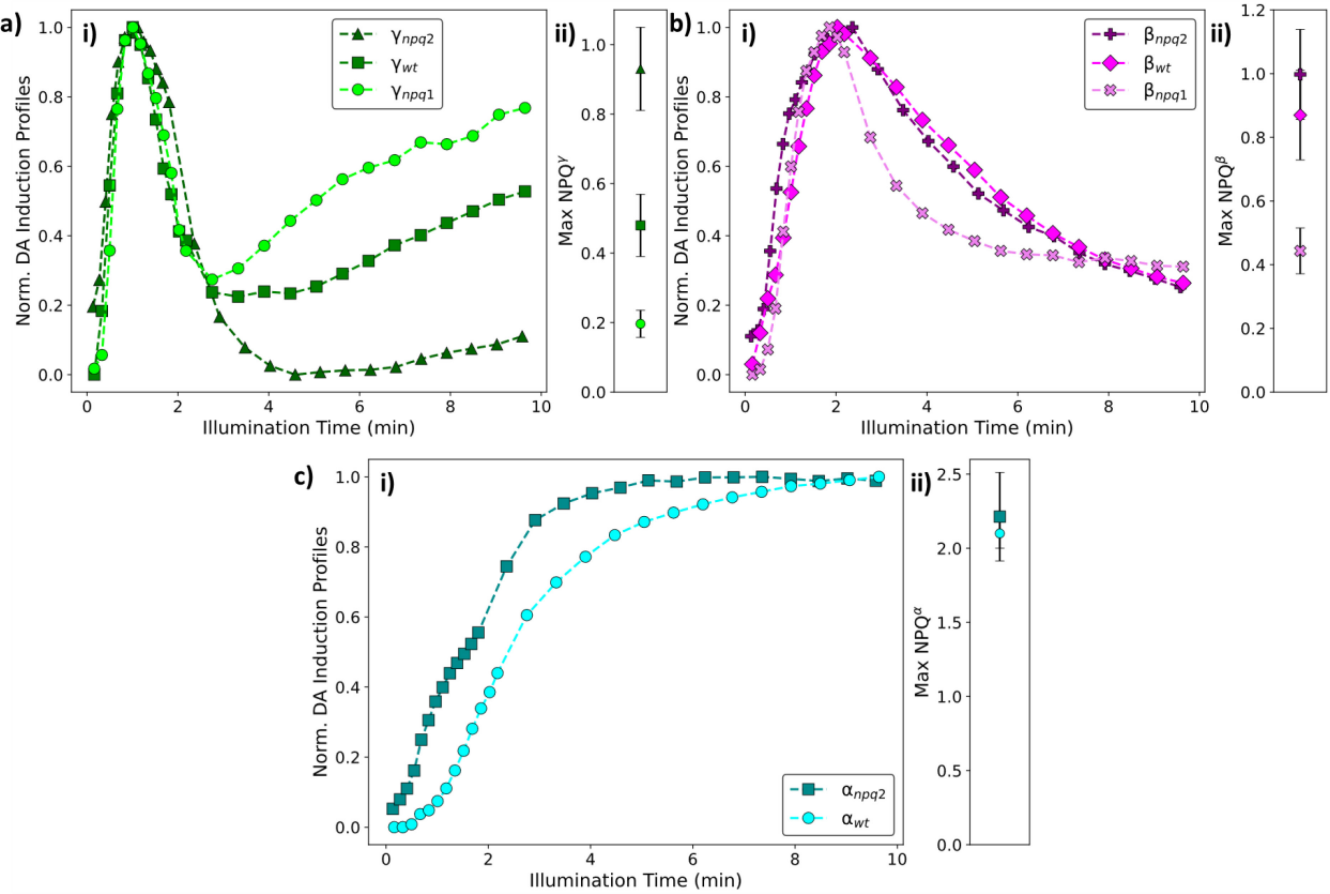
Comparison between the induction profiles (i) and the maximum NPQ contributions (ii) of (a) the γ, (b) the β components for the dark‐adapted NPQ induction curves of *npq1*, wt and *npq2* *A. thaliana* . (c) The α components for the dark‐adapted NPQ induction curves of wt and *npq2* *A. thaliana* .

To understand the role(s) of Zx in NPQ induction, the components of *npq1* (Zx‐lacking), wt and *npq2* (Zx‐rich) plants were directly compared (Figure 6).

Both the β and γ components contribute to the dark‐adapted NPQ induction dataset for the *npq2*, wt and *npq1* plants. These two components are thought to be associated with NPQ triggered by the protonation of PsbS upon illumination (Ramakers et al. 2025). The induction profile of the γ component is broadly similar in these three different genotypes, at least for the first 2 min. In all three genotypes explored the initial increase and the local NPQ maximum of this component are very similar (Figure 6a(i)). The maximum contributions of this component to the overall NPQ (Figure 6a(ii)) were found to mirror the level of Zx present (e.g., *npq1* < wt < *npq2*). For the β component, this comparison again reveals that the induction profile is almost identical in all three genotypes (Figure 6b(i)). Additionally, the maximum contribution of this component to the overall NPQ (Figure 6b(ii)) also mirrored the amount of Zx present in the leaves (e.g., *npq1* < wt < *npq2*). Together, this reveals that these PsbS‐dependent components, exhibit induction kinetics which are Zx‐independent, but the presence of Zx amplifies the amplitude of these PsbS‐dependent components. Comparing *npq2* (100% Zx) and *npq1* (0% Zx) reveals Zx‐induced amplifications of ~2.5‐ and ~4.6‐fold for the β and γ components, respectively. This is somewhat reminiscent of the results of a previous study which used ΔpH/qE‐titrations to show that Zx acted as a pH‐sensitiser for qE (Pérez‐Bueno et al. 2008).

In addition to amplifying the effect of PsbS, Zx also contributes a separate component to NPQ in wt and *npq2* plants (Figure 6c(i)), which is absent in *npq1* plants. In wt the α component is associated with the accumulation of Zx via the xanthophyll cycle (Ramakers et al. 2025). Previously in wt plants, a simplified model of the xanthophyll cycle showed that the induction profile of this component could well be explained by a model including an additional interaction step, indicating that Zx requires an additional interaction (partner) to be present to contribute to the overall NPQ. This model explained the profile of this component in wt and is broadly consistent with other studies, which have suggested that Zx‐dependent quenching follows an allosteric model (Rees et al. 1989; Noctor et al. 1991; Ruban et al. 1993; Ruban and Horton 1995, 1999; Horton et al. 2000, 2008; Johnson et al. 2008, 2009; Horton 2012). Whilst the maximum contribution of this component is similar in both genotypes (Figure 6c(ii)), the presence of Zx in dark‐adapted leaves (*npq2*) leads to the loss of the distinctive lag‐time and possibly slightly faster induction kinetics of the α component. Interestingly, the α component does not contribute to either of the *npq4* (PsbS‐lacking) NPQ induction datasets (Figure 5). Since the xanthophyll cycle is known to be fully functional in the *npq4* plants, this indicates that the loss of PsbS leads to the loss of the Zx quenching interaction partner. This demonstrates that either the Zx quenching interaction partner is PsbS itself or a ‘species’ which can only form in the presence of protonated PsbS. This also seems to be consistent with the faster kinetics of the light‐adapted $\alpha_{\mathrm{npq}2}^{\prime }$ component (Figure 3b(i)). This distinct change mirrors the faster induction kinetics of $\beta_{\mathrm{npq}2}^{\prime }$ and $\gamma_{\mathrm{npq}2}^{\prime }$ components and reveals that a portion of the ‘free’‐Zx pool in *npq2* engages in quenching as soon as additional interaction partners are formed upon further illumination. Together, these results show that Zx plays two distinct roles in the induction of NPQ, namely as an amplifier for the PsbS‐dependent NPQ components and by providing an additional Zx‐dependent quenching component, which becomes the dominant source of NPQ over illumination time, and in turn requires the presence of PsbS. This seems to be inconsistent with the idea in the literature where the Zx‐dependent and Zx‐independent phases of NPQ induction arise from the same underlying molecular mechanism (Johnson et al. 2009).

### 4.2 Effects of Light‐Adaptation on the NPQ Induction Components

**FIGURE 7:**
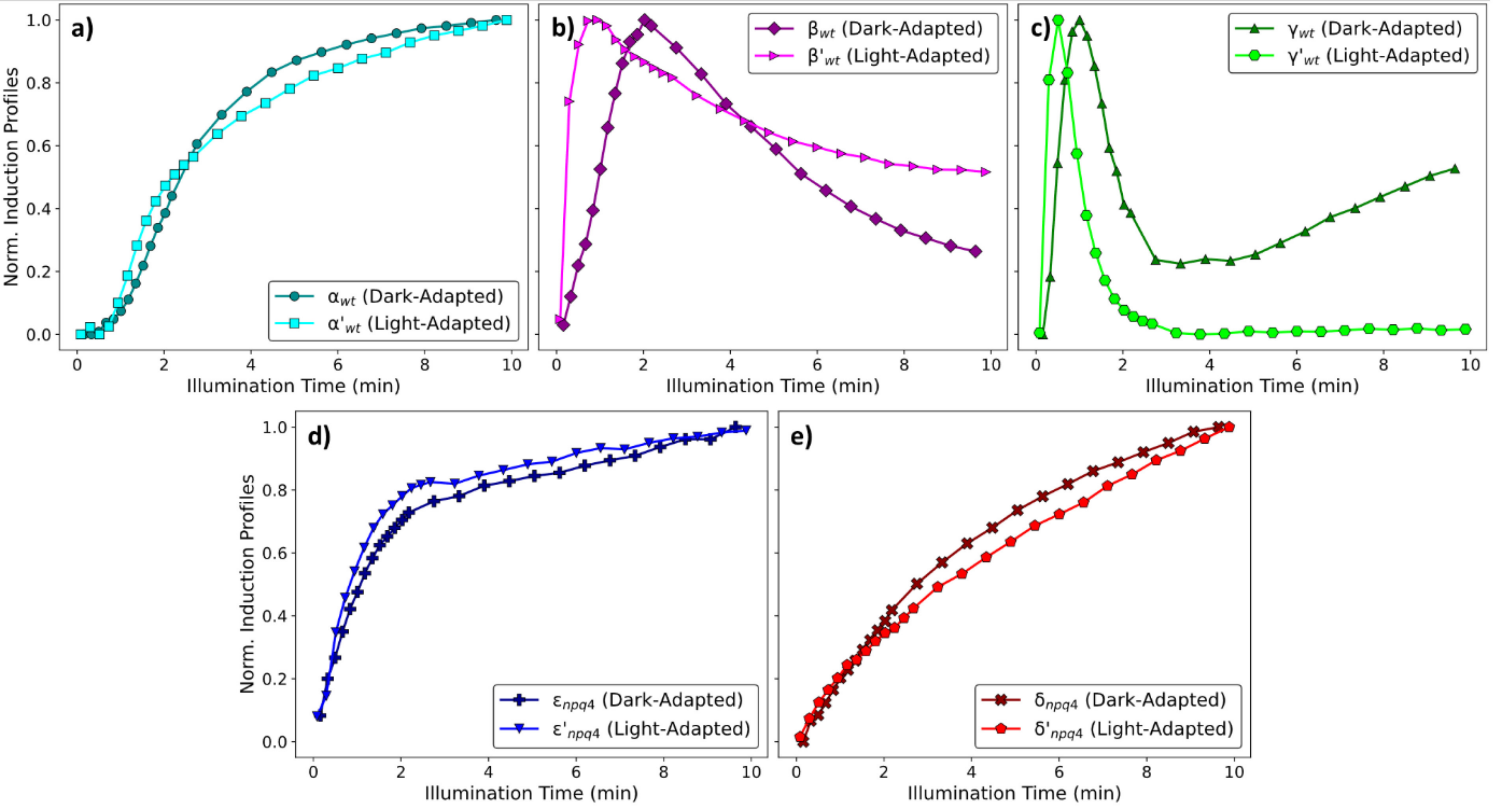
Comparison between the normalised induction profiles associated with the dark‐ and light‐adapted (a) α, (b) β, (c) γ, (d) ε and (e) δ NPQ components. The contributions of these components to the associated NPQ induction curves are shown in Figure 2 (α, β and γ) and Figure 5 (ε and δ).

A comparison between the dark‐ and light‐adapted induction profiles of the α component, in plants with a fully functional xanthophyll cycle, reveals that this component is largely unaffected by light‐adaptation (Figure 7a), although it should be kept in mind that this component is only significantly induced at actinic light intensities above ~200 μmol/m^2^ s. Furthermore, similar comparisons also show that both the ε (Figure 7d) and δ (Figure 7e) components are largely unchanged by light‐adaptation.

Contrasting this, the induction kinetics of the β (Figure 7b) and γ (Figure 7c) components are significantly faster following light‐adaptation in *npq2*, wt and *npq1* plants. Both of these components are thought to arise due to the protonation of PsbS upon the acidification of the lumenal space (Ramakers et al. 2025). The differences between the kinetic profiles of the β and γ components are assumed to be due to the effects of the K^+^‐efflux antiporter (Armbruster et al. 2014, 2016) and will be further explored in a subsequent experimental study. Interestingly, the similarity in the profiles of these components and the changes they undergo following light‐adaptation in all three genotypes (Figure 6, Figure S10) shows that these changes in the induction kinetics of the β and γ components are completely independent of Zx, whereas the amplitudes are highly Zx‐dependent. This is also supported by the similarity between the dark‐adapted profiles of the $\beta_{\mathrm{npq}2}$ and $\gamma_{\mathrm{npq}2}$ components and their *npq1* and wt counterparts (Figure 6a,b). The similarity of the profiles of the dark‐ and light‐adapted β and γ components in wt, *npq2* and *npq1* plants (Figure 6, Figure S10) seems to be inconsistent with the idea that the same molecular mechanism underpins both the zeaxanthin‐independent and zeaxanthin‐dependent qE, as the profile of their induction curves is unaffected by differing levels of Zx present upon induction (Johnson et al. 2009; Horton 2012). *npq4*, which lacks PsbS, is the only genotype which did not show faster NPQ induction kinetics following light‐adaptation (Figure 5). Since the CO_2_ assimilation rates of wt and *npq4* are known to be the same during a series of different light intensity steps (Schiphorst et al. 2023), it can be concluded that the light‐adapted changes in the activity of the CBB cycle are unaffected by the loss of PsbS. This implies that the change in the induction kinetics of NPQ following light adaptation is solely due to a PsbS‐related process.

## 5 Conclusions

In conclusion, our multivariate analysis pipeline has revealed that both dark‐ and light‐adapted NPQ induction can be explained by several components exhibiting distinct induction kinetics (α, β, γ, δ and ε). These different components are associated with Zx (α), the protonation of PsbS (β and γ), photo‐inhibition (δ) and the acidification of the lumen in PsbS‐lacking plants (ε). The empirical difference between the dark‐adapted induction curves of these genotypes can be explained by changes in the relative contributions of these components. Comparing the components and their contributions to the dark‐adapted NPQ induction curves for *npq2*, wt and *npq1* plants reveals that Zx acts both as an amplifier for the PsbS‐dependent components as well as providing its own Zx‐dependent quenching component (in *npq2* and wt), that in turn requires the presence of PsbS. The lack of a contribution of the α component to the NPQ induction of *npq4* plants revealed that the previously postulated Zx‐quenching interaction partner is likely a ‘species’ which can only form in the presence of PsbS (Ramakers et al. 2025). Overall, this analysis reveals several different synergistic interactions between Zx and PsbS. First, Zx amplifies the PsbS‐dependent components and the presence of PsbS allows Zx to also participate fully in NPQ. Additionally, the PsbS‐dependent quenching (β and γ) provides a high degree of transient NPQ before the Zx‐dependent quenching (α) builds up and becomes the dominant source of quenching over time. Directly comparing the normalised induction profiles of the identified NPQ components revealed that the faster NPQ induction kinetics seen upon light‐adaptation are predominantly driven by the significantly faster kinetics of the β and γ components. The induction profiles of all of the other identified components (α, ε and δ) were found to remain largely unaffected by light‐adaptation of the leaf. Interestingly, the β and γ components exhibit significantly faster induction kinetics in the light‐adapted state for wt, *npq2* and *npq1 A. thaliana*, indicating that, while Zx has a significant effect on the overall amplitude of these components, the faster light‐adapted induction kinetics of these components are unrelated to the degree of Zx accumulation during light‐adaptation. This demonstrates that the faster NPQ induction seen in light‐adapted plants is solely due to changes in the induction kinetics of the PsbS‐dependent quenching processes.

## Author Contributions

L.A.I.R. and H.v.A. conceived the project. L.A.I.R. collected the data and performed the analysis. L.A.I.R. designed the experimental methodology and novel analysis pipeline, with substantial inputs from J.H. and H.v.A. L.A.I.R. and H.v.A. wrote the first draft of the manuscript, with substantial inputs from J.H. All authors contributed to the manuscript.

## Conflicts of Interest

The authors declare no conflicts of interest.

## Supporting information


**Figure S1.** Supporting Information.

## Data Availability

The original contributions presented in the study, as well as the analysis software package used (Yggdrasill_App.exe) are available to download from a GitHub repository: L‐Ramakers/Yggdrasill.
