# Supplementary material for: A Novel Multivariate Analysis: Overturning Long‐Held Beliefs About Non‐Photochemical Quenching
Source: Physiol Plant. 2025 Jul 19;177(4):e70420. doi: 10.1111/ppl.70420 (PMC12275998; doi:10.1111/ppl.70420)
Supplement: Supplementary file 1 — Figure S1. Supporting Information. [file PPL-177-e70420-s001.pdf]

A Novel Multivariate Analysis: Overturning long-held beliefs about non-photochemical quenching

Lennart A. I. Ramakers[1], Jeremy Harbinson[1] and Herbert van Amerongen[1]*

1 – Laboratory of Biophysics, Wageningen University, Wageningen, The Netherlands

*Correspondence

Herbert van Amerongen

E-mail: herbert.vanamerongen@wur.nl

Supplementary Information

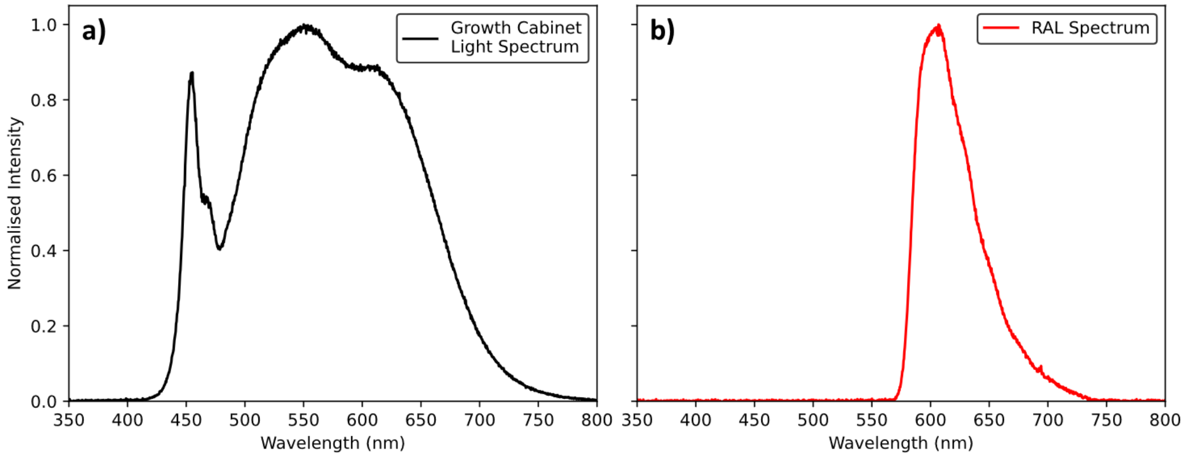

Figure S1: Emission spectra of a) the actinic light utilised by the Hettich ESP PRC 1200 WL growth cabinet and b) the red actinic light used in the PAM measurements.

Notes S1 – Multivariate Analysis Pipeline Summary

The multivariate analysis pipeline was developed to identify the kinetically distinct induction components underpinning an NPQ induction dataset and deconvolute the dataset using the identified components. Developed using wild-type and *npq1 A. thaliana* NPQ induction datasets (Ramakers *et al.*, 2025), this pipeline consists of 3 steps.

1. Principal Component Analysis (PCA) is a multivariate technique which can be used to identify and separate different sources of variation within mean-centred datasets (Maćkiewicz and Ratajczak, 1993; Fritzsch *et al.*, 2018). Here, it is used to explore the variance within the NPQ induction dataset, identify the minimum number of principal components which can explain this variance and extract the induction profiles of these underlying components. This allows the components which vary strongly, with respect to the leaf-to-leaf biological variance, to be identified. In the case of wild-type *A. thaliana*, PCA reveals that the variance can be adequately described by 2 kinetically distinct components, revealing there are 2 strongly varying components underpinning the NPQ induction dataset (Ramakers *et al.*, 2025).

2. Following PCA, full harmonic phasor analysis (FH-PhA) is applied to the dataset (Bader *et al.*, 2014; Franssen *et al.*, 2020). This technique can be used to explore changes in the profiles of curves within a dataset and in our analysis pipeline this technique is used to identify any components which vary weakly, with respect to the leaf-to-leaf biological variance, over the dataset. Basically, applying FH-PhA allows each curve to represented as a point in a 2D (phasor) space where the exact position of the point is dictated by the profile of the curve. Applying this to the entire dataset as well as the PCA identified components yields a data cloud of points in this 2D (phasor) space, with the shape of this cloud indicating the number of underlying components (*e.g.* a linear cloud indicates that there are 2 components, a triangular cloud indicates there are 3 components *etc*.). Interestingly, the PCA identified components are always found to bound part of the data cloud (Ramakers *et al.*, 2025). If the data cloud is only partially bounded by the PCA identified components, the other bound of the data cloud can be extrapolated using the positions of the PCA components and the centroid of the cloud. In this way weakly varying components can be identified. Finally, using linear algebra each point in the data cloud can be expressed as a sum of the PCA and extrapolated components allowing an approximate contribution of each of these components to the curves in the dataset to be calculated.
3. Finally, the results of PCA and FH-PhA are used to guide the non-negative matrix factorisation (NMF) of the NPQ induction dataset (Berry *et al.*, 2007). Specifically, the results of PCA and FH-PhA are used provide an initial estimate of the contribution of each component to the dataset for the NMF step. Both the induction profiles of the components and their contribution to the overall NPQ are optimised by the NMF step (*via* the ALS algorithm).

Taken together, this 3 step multivariate analysis pipeline allows any NPQ induction dataset to be deconvolved into its underlying components, based on their distinct induction kinetics. Following this deconvolution the molecular processes underpinning the obtained components can be identified using measurements performed on chemically treated leaves (*e.g.* using DTT, nigericin and DCMU) (Ramakers *et al.*, 2025).

Notes S2 – Long-term F_m quenching Correction

Long-term quenching can either be identified by a low F_m value (which should be NPQ free in full dark-adapted plants) or by a short PSII fluorescence lifetime (generally expected to be ~2 ns in healthy dark-adapted plants) (Belgio *et al.*, 2012). The fluorescence lifetime of PSII can be obtained from ultrafast fluorescence measurements once the data has been deconvoluted into its PSI and PSII contributions. This is summarised in Figure S2.

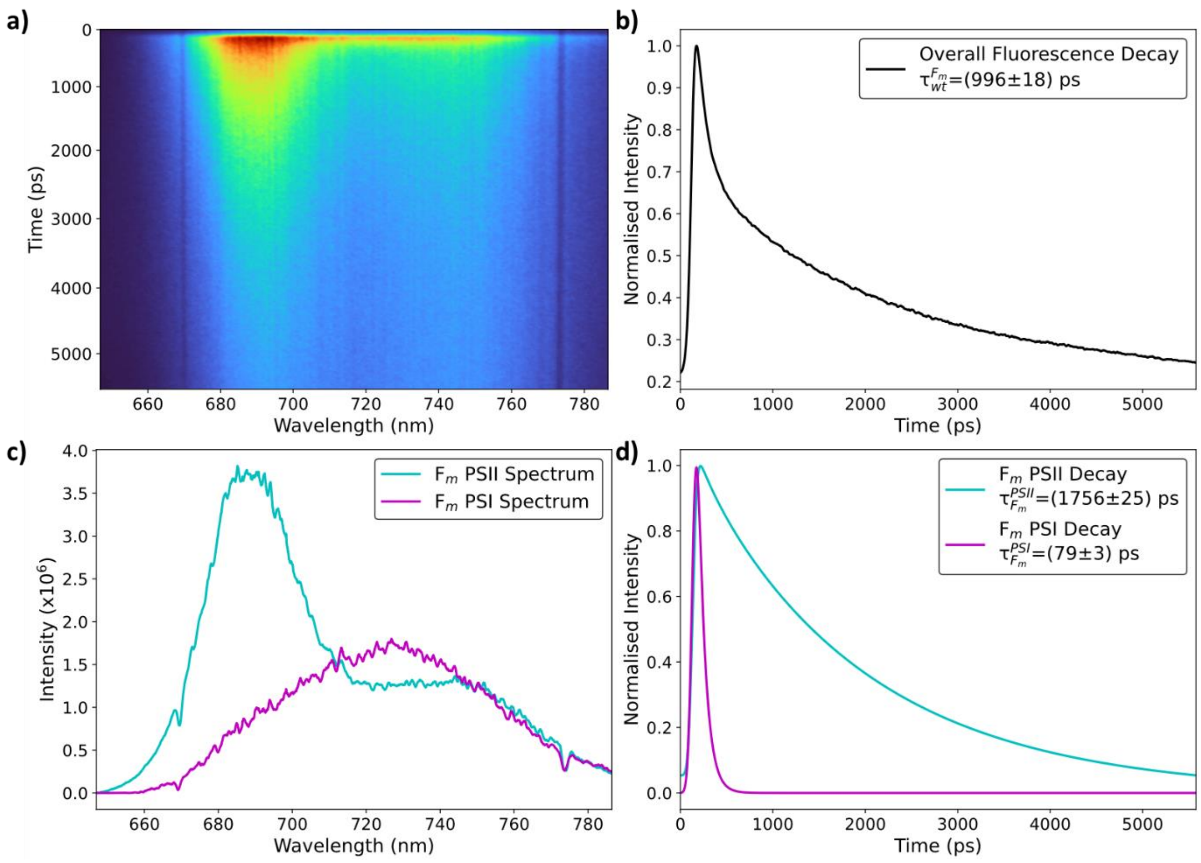

Figure S2: A representative a) F_m ultrafast fluorescence spectrum and b) the associated overall fluorescence decay curve for wt dark-adapted *A. thaliana*. The deconvoluted c) average F_m PSII (cyan) and PSI (magenta) spectra (n = 3) and d) their associated average PSII (cyan) and PSI (magenta) decay curves (n = 3), for wt dark-adapted *A. thaliana*.

A correction factor for this sustained quenching can be derived by equating the yield and lifetime forms of the Stern-Volmer equation:

$$\frac{F_m^{No\,NPQ} - F_m^{Observed}}{F_m^{Observed}} = \frac{\tau_{F_m}^{No\,NPQ} - \tau_{F_m}^{Observed}}{\tau_{F_m}^{Observed}} \; (= NPQ) \quad (1)$$

Where $F_m^{No\,NPQ}$ is the theoretical value of the dark-adapted fluorescence yield with fully closed PSII RCs, $F_m^{Observed}$ is the measured value of the dark-adapted fluorescence yield with fully closed PSII RCs with long-term quenching, $\tau_{F_m}^{No\,NPQ}$ is the theoretical value of the dark-adapted fluorescence lifetime with fully closed PSII RCs, $\tau_{F_m}^{Observed}$ is the measured value of the dark-adapted fluorescence lifetime with fully closed PSII RCs with long-term quenching and NPQ is non-photochemical quenching.

This can be rearranged to obtain the following expression for $F_m^{No\,NPQ}$:

$$F_m^{No\,NPQ} = F_m^{Observed}\left[\frac{\tau_{F_m}^{No\,NPQ} - \tau_{F_m}^{Observed}}{\tau_{F_m}^{Observed}} + 1\right] \quad (2)$$

This expression can be simplified to obtained the following expression:

$$F_m^{No\ NPQ} = F_m^{Observed} \left[\frac{\tau_{F_m}^{No\ NPQ}}{\tau_{F_m}^{Observed}} \right] \quad (3)$$

The PSII-only lifetime obtained from fully dark-adapted and DCMU infiltrated wt *A. thaliana* leaves is (1753 ± 9) ps and so the corresponding overall average lifetime obtained from this data ((1034 ± 9) ps) was used to calculate the correction factors for the other mutant genotypes measured (*e.g.* $\tau_{F_m}^{No\ NPQ} = \tau_{wt}^{DCMU}$). The lifetimes and correction factors are summarised in Figure S3.

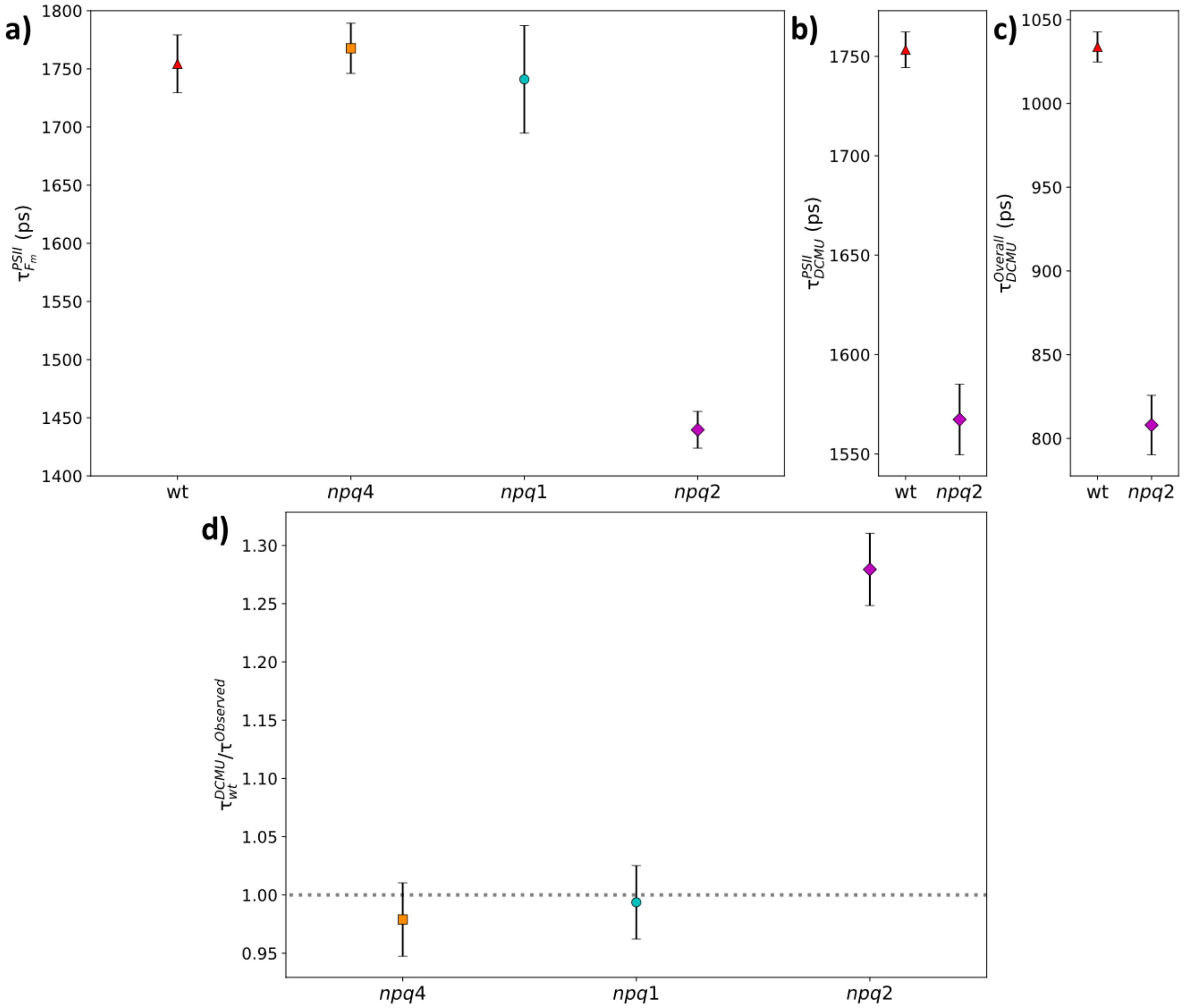

Figure S3: a) Average (n = 3) PSII only lifetimes for wt, *npq4*, *npq1* and *npq2 A. thaliana* plants; Average (n = 3) b) PSII-only lifetimes and c) overall lifetimes, for DCMU infiltrated wt and *npq2 A. thaliana* leaves. d) Average (n = 3) correction factors for *npq4*, *npq1* and *npq2 A. thaliana* plants calculated using the overall lifetimes. All plants were dark-adapted overnight prior to measurement.

Here, the PSII-only lifetimes ($\tau_{F_m}^{PSII}$) were used to determine if any of the studied genotypes exhibited long-term quenching (*eg.* $\tau_{F_m}^{PSII} \ll 1750$ ps) and the overall lifetimes ($\tau^{Overall}$) were used to calculate

the correction factor for the PAM data to account for the fluorescence yield contribution of PSI. Long-term NPQ in the dark-adapted F_m state was only observed for *npq2* plants yielding an average correction factor of (1.28 ± 0.03).

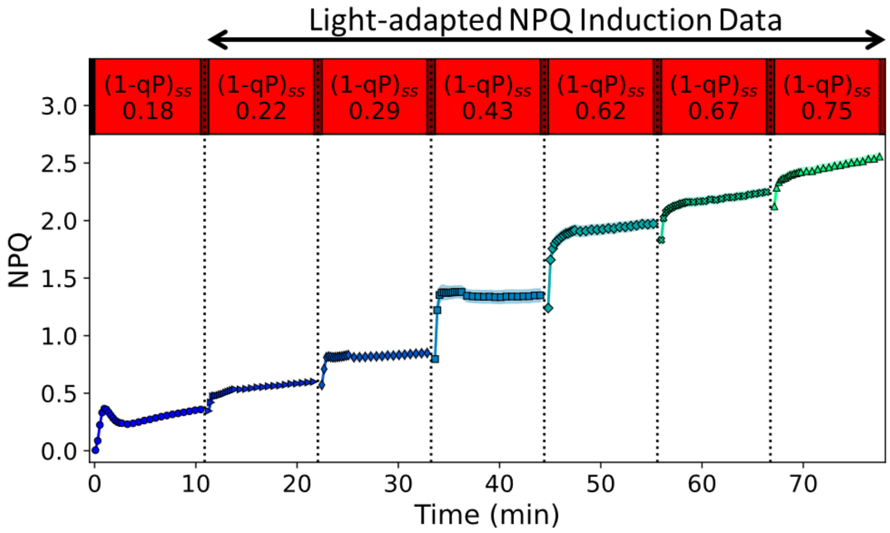

Figure S4: NPQ induction curves for wt *A. thaliana* obtained during a light response curve measurement employing a series of steps at higher actinic light intensities, leading to increasing values of $(1\text{-qP})_{ss}$. Each curve is the average NPQ induction of individual measurements carried out on separate leaves (n = 5), the shaded area shows the associated standard error.

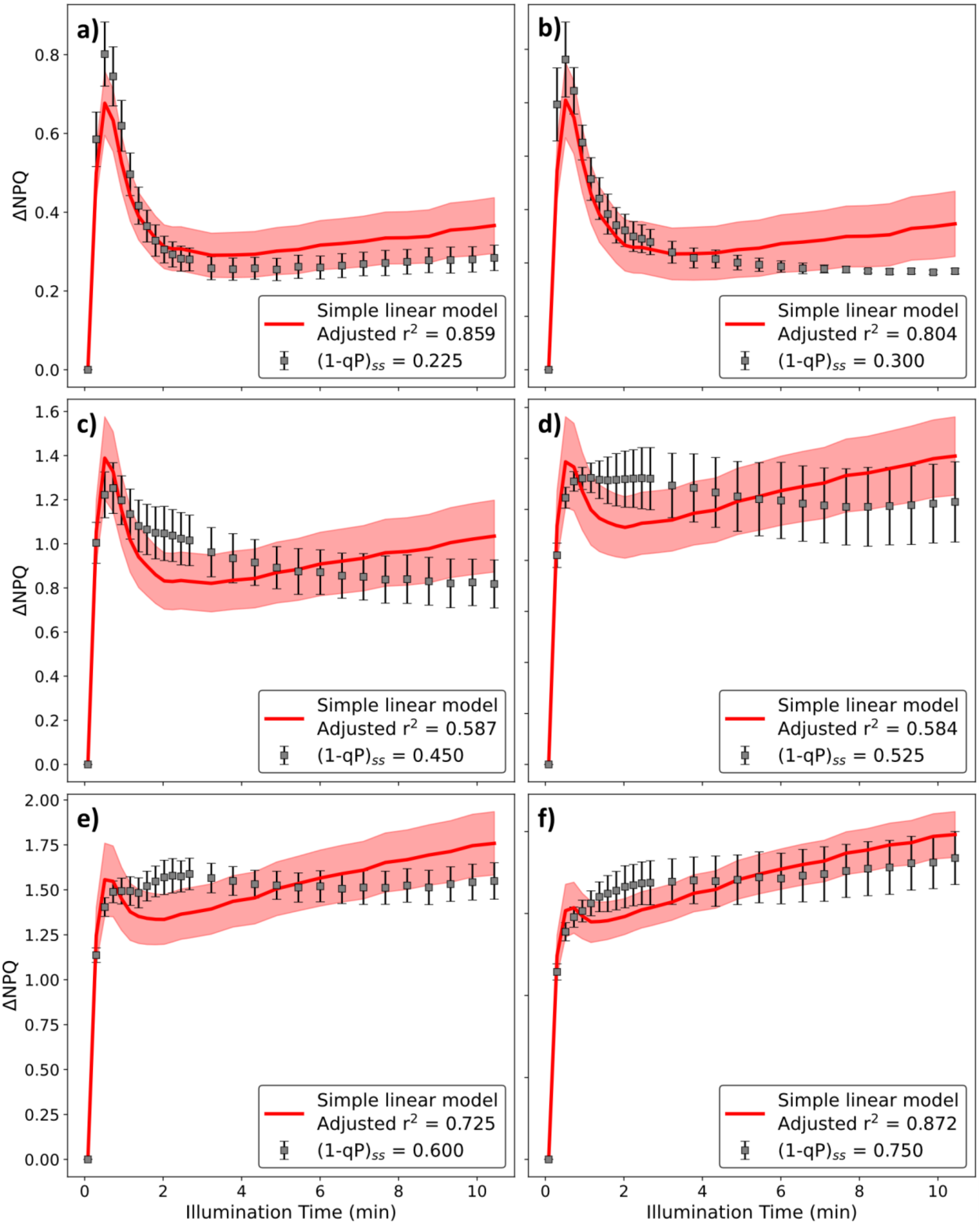

Figure S5: Representative (mis)fits of the simple linear-combination model (red), using the lowest and highest light induction curves in the dataset, for the light-adapted NPQ induction curves at $(1\text{-qP})_{ss}$ values of a) 0.225, b) 0.3, c) 0.45, d) 0.525, e) 0.6 and f) 0.75 for wild-type (wt) *A. thaliana* including adjusted r^2 values for each fit. Error bars showing the measurement standard error and the shaded area show the standard error associated with the simple linear-combination fit.

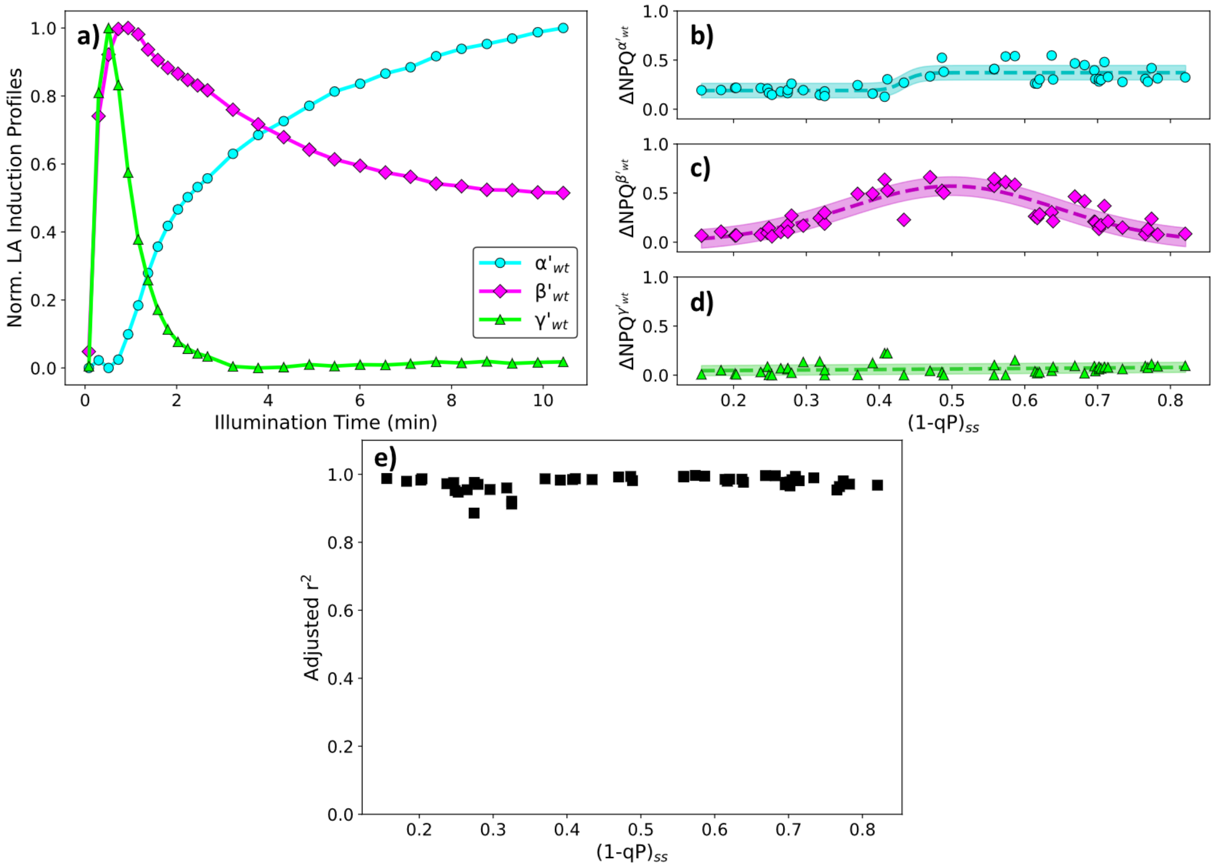

Figure S6: a) Normalised ΔNPQ components for light-adapted wt *A. thaliana* and their contribution to the overall ΔNPQ b) α'_{wt}, b) β'_{wt} and c) γ'_{wt}, respectively. e) The adjusted r^2 values obtained for the fits of the light-adapted components to the wt light response curve ΔNPQ dataset. All plants were illuminated using red actinic light.

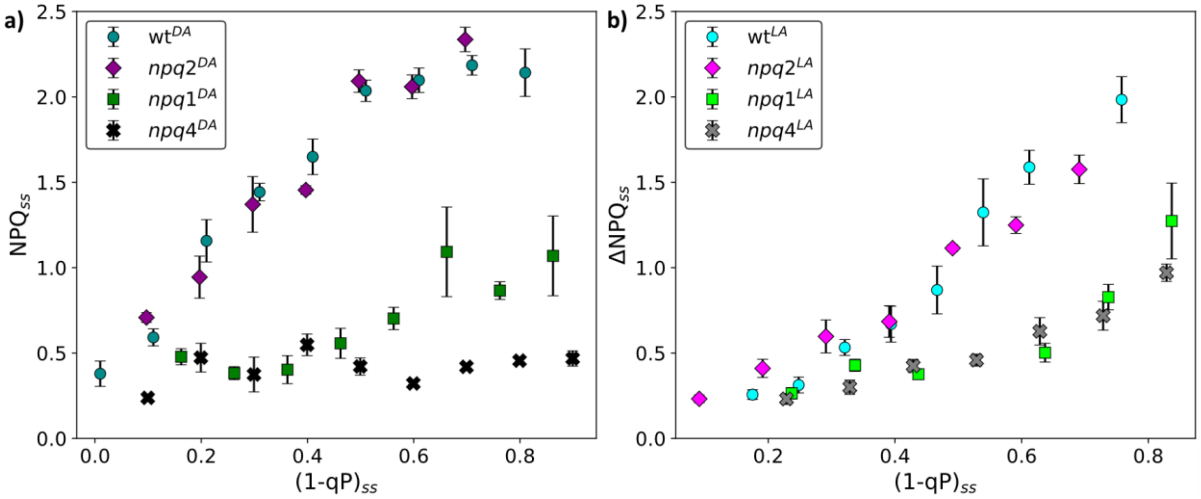

Figure S7: Comparison between the steady-state NPQ values (NPQ_{ss}) versus $(1\text{-qP})_{ss}$ for a) dark- and b) light-adapted wt, *npq2*, *npq1* and *npq4 A. thaliana*.

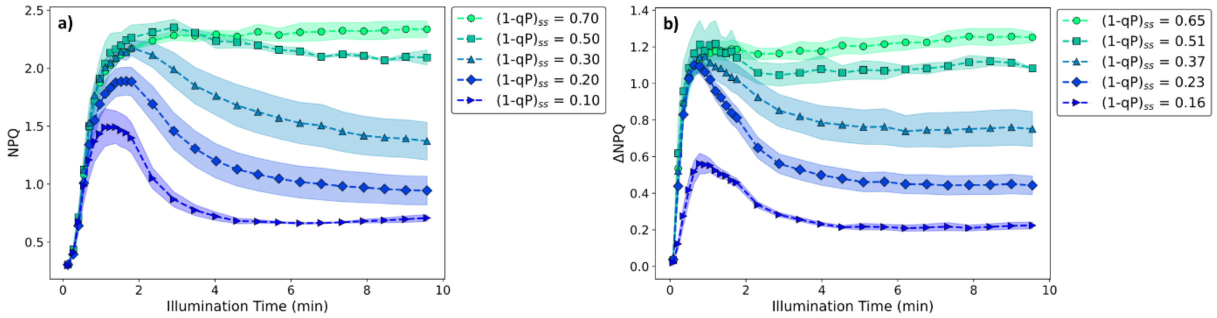

Figure S8: *npq2 A. thaliana* a) NPQ and b) ΔNPQ induction curves obtained at a range of different actinic light intensities, leading to differing values of $(1\text{-qP})_{ss}$. Each curve is a binned average of individual measurements carried out on separate leaves, the shaded area shows the associated standard error.

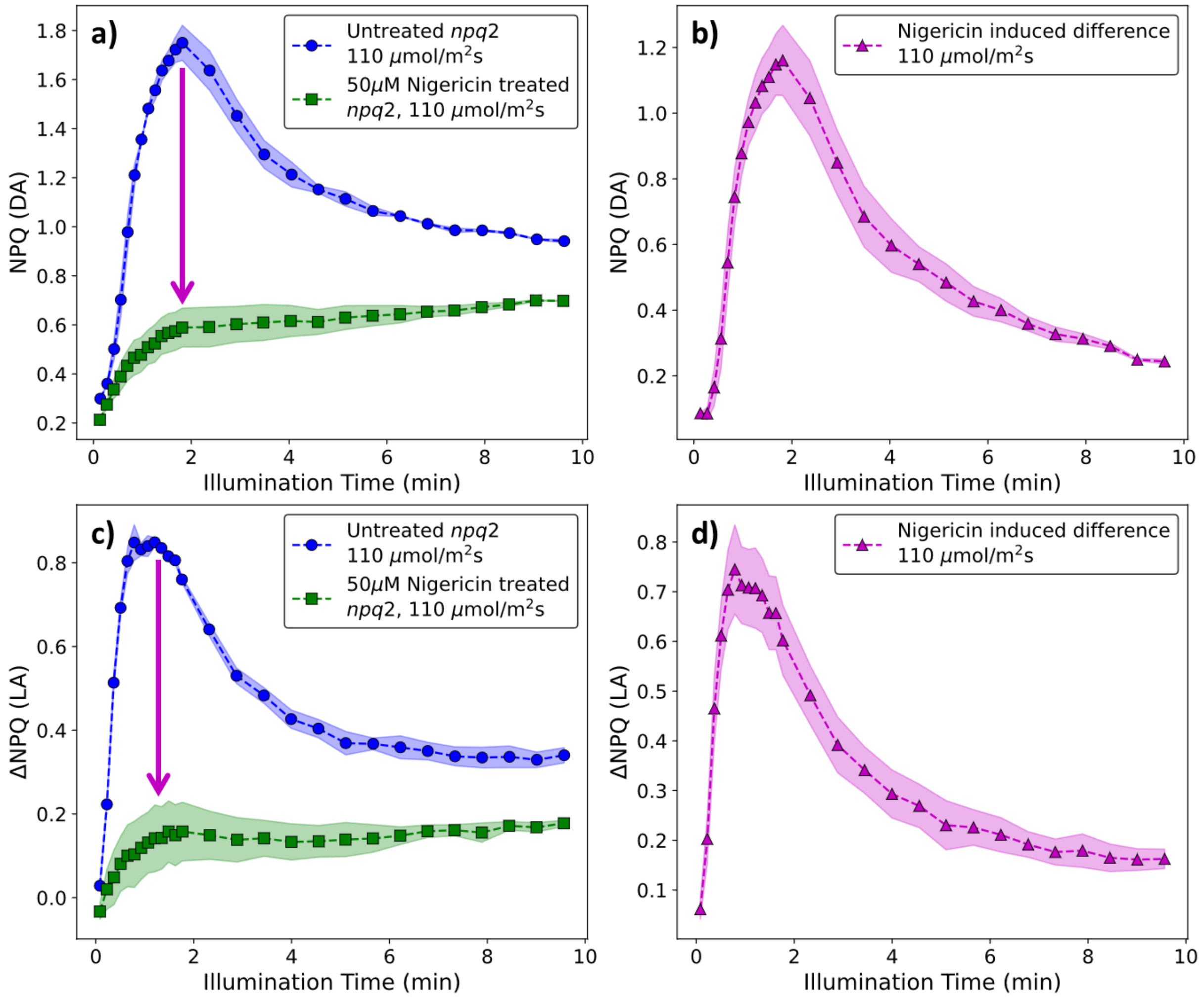

Figure S9: a) Dark-adapted NPQ induction curves for untreated (blue circles) and 50 µM nigericin treated (green squares) *npq2 A. thaliana* leaves, obtained for illumination at an actinic light intensity of 110 µmol/m^{2}s, and b) the dark-adapted nigericin-induced NPQ difference curve (magenta triangles); c) Light-adapted ΔNPQ induction curves for untreated (blue circles) and 50 µM nigericin treated (green

squares) *npq2 A. thaliana* leaves, obtained for illumination at an actinic light intensity of 110 µmol/m^{2}s, and d) the light-adapted nigericin-induced ΔNPQ difference curve (magenta triangles). Shaded areas show the associated standard error for each measurement.

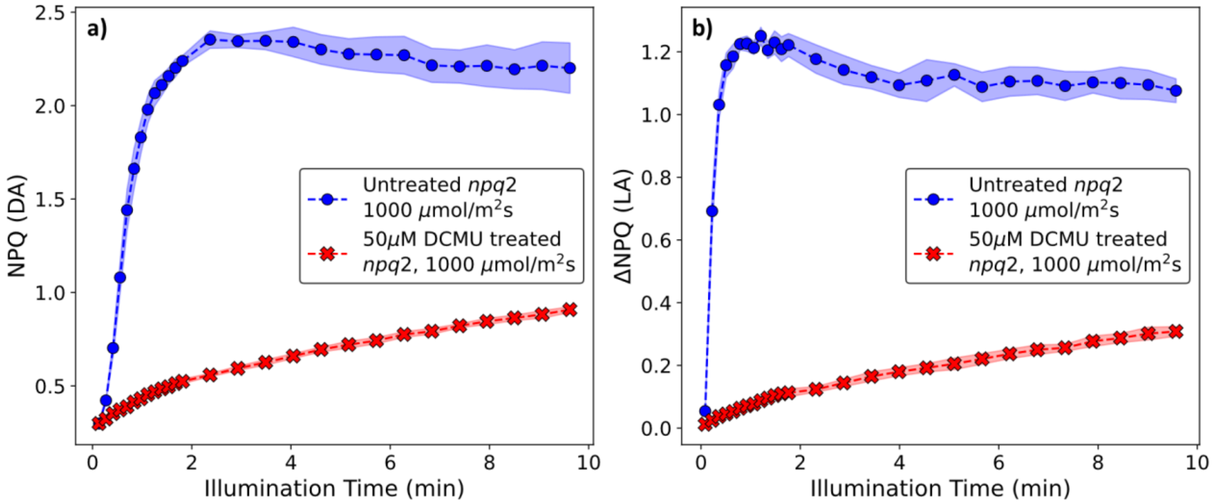

Figure S10: a) Dark-adapted NPQ induction curves for untreated (blue circles) and 50 µM DCMU treated (red crosses) *npq2 A. thaliana* leaves, obtained for illumination at an actinic light intensity of 1000 µmol/m^{2}s; b) Light-adapted ΔNPQ induction curves for untreated (blue circles) and 50 µM DCMU treated (red crosses) *npq2 A. thaliana* leaves, obtained for illumination at an actinic light intensity of 1000 µmol/m^{2}s. Shaded areas show the associated standard error for each measurement.

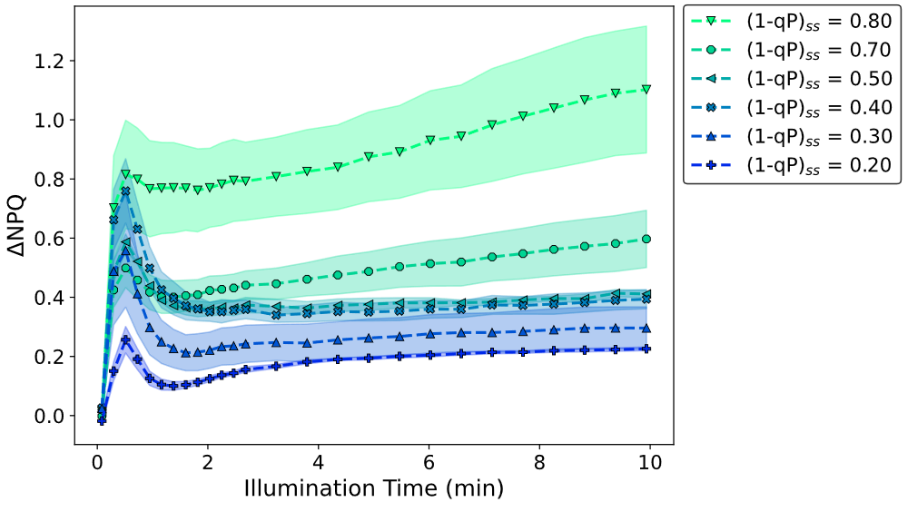

Figure S11: *npq1 A. thaliana* ΔNPQ induction curves obtained at a range of different actinic light intensities, leading to differing values of $(1\text{-qP})_{ss}$. Each curve is a binned average of individual measurements carried out on separate leaves, the shaded area shows the associated standard error.

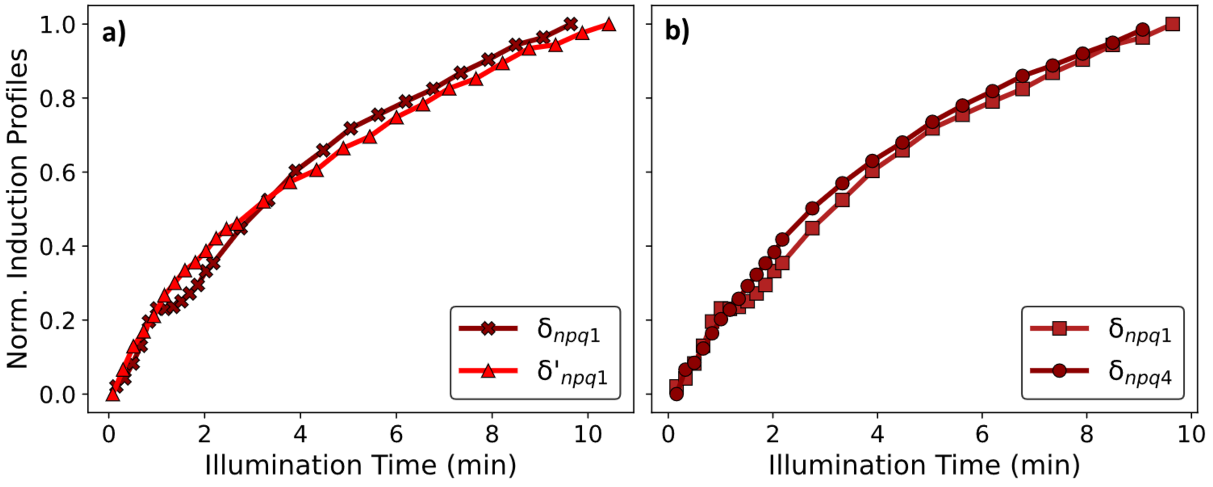

Figure S12: Comparison between a) the δ_{npq1} (crosses; dark-adapted) and δ'_{npq1} (triangles; light-adapted) components and between b) the δ_{npq1} (squares; dark-adapted) and δ_{npq4} (circles; dark-adapted) components. The contributions of these components to the associated NPQ induction curves are shown in figures 3 (*npq1*) and 4 (*npq4*).

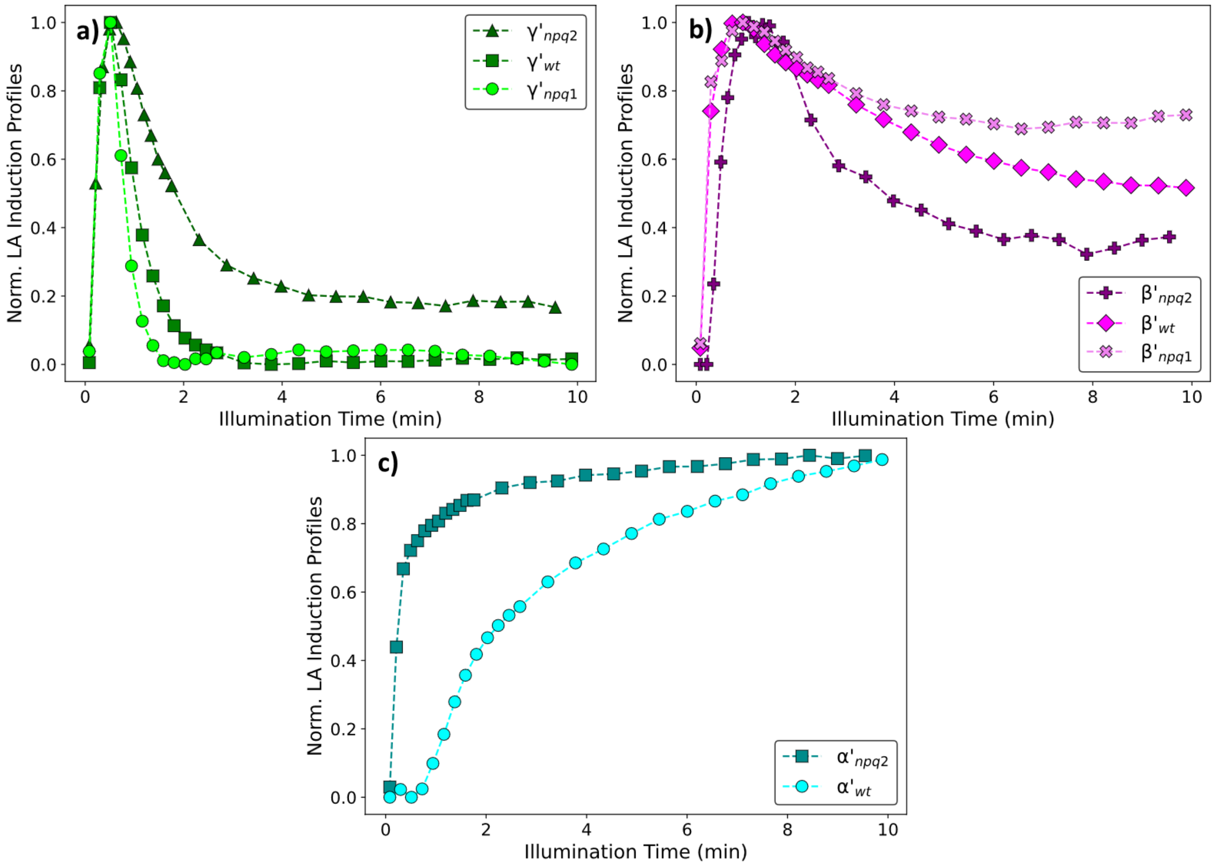

Figure S13: Comparison between the light-adapted a) γ_{npq2}, γ_{wt} and γ_{npq1}, b) β_{npq2}, β_{wt} and β_{npq1} and c) α_{npq2} and α_{wt}, components for *npq2*, wt and *npq1 A. thaliana*. The contributions of these components to the associated NPQ induction curves are shown in figures 2 (wt), 3 (*npq2*) and 4 (*npq1*).

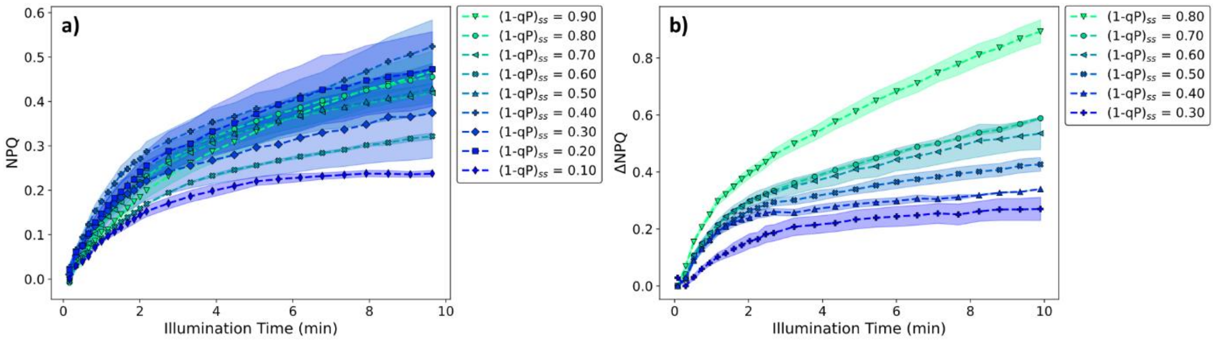

Figure S14: *npq4 A. thaliana* a) NPQ and b) ΔNPQ induction curves obtained at a range of different actinic light intensities, leading to differing values of $(1\text{-qP})_{ss}$. Each curve is a binned average of individual measurements carried out on separate leaves, the shaded area shows the associated standard error.

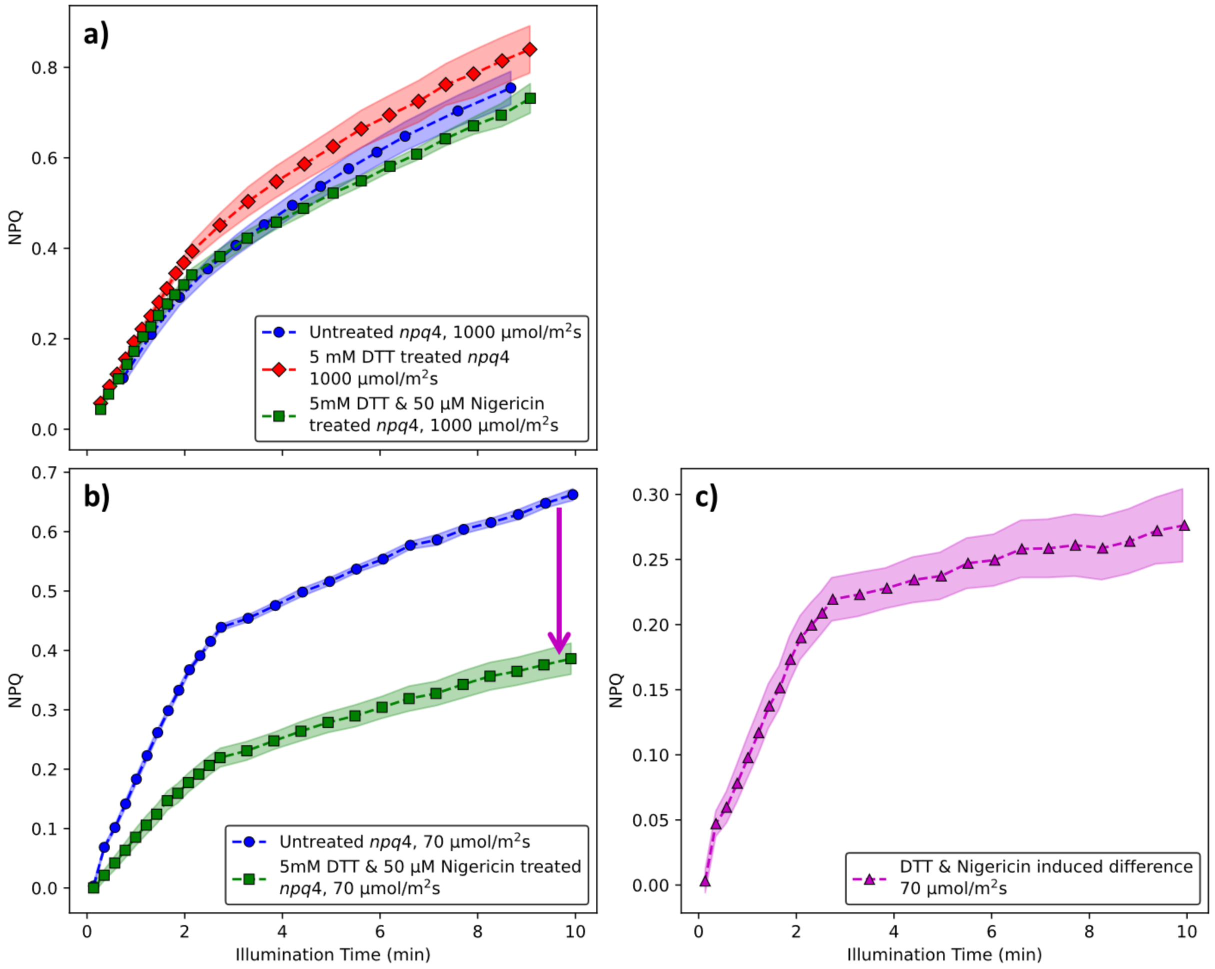

Figure S15: a) NPQ induction curves for untreated (blue circles), 5 mM DTT treated (red diamonds) and 5 mM DTT & 50 μM nigericin treated (green squares) *npq4 A. thaliana* leaves, obtained for illumination at an actinic light intensity of 1000 μmol/m^{2}s. b) NPQ induction curves for untreated (blue circles) and 5 mM DTT & 50 μM nigericin treated (green squares) *npq4 A. thaliana* leaves, obtained for illumination at an actinic light intensity of 70 μmol/m^{2}s, and c) the (DTT & nigericin)-induced NPQ

difference curve (magenta triangles). Shaded areas show the associated standard error for each measurement.

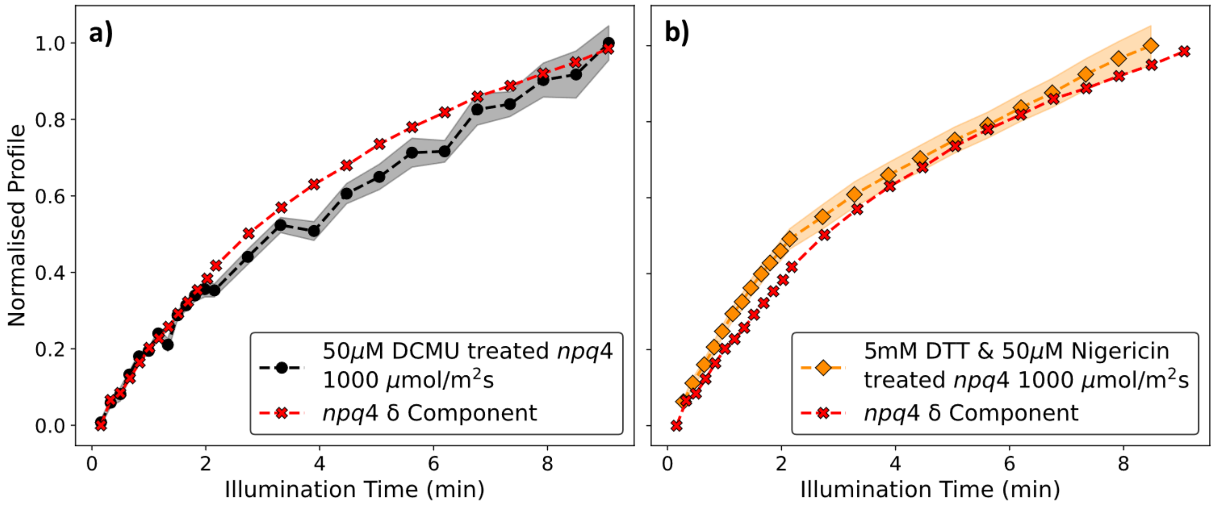

Figure S16: a) Comparison between the *npq4 A. thaliana δ* component (red crosses; dark-adapted) and the NPQ induction curves for 50 µM DCMU treated leaves (black circles). b) Comparison between the *npq4 A. thaliana δ* component (red crosses; dark-adapted) and the NPQ induction curves for 5 mM DTT & 50 µM nigericin treated leaves (dark orange triangles). Shaded areas show the associated standard errors.

References

Bader, A.N. *et al.* (2014) 'Phasor approaches simplify the analysis of tryptophan fluorescence data in protein denaturation studies', *Methods and Applications in Fluorescence*, 2(4), p. 045001. Available at: https://doi.org/10.1088/0000-0000/2/4/045001.

Belgio, E. *et al.* (2012) 'Higher plant photosystem II light-harvesting antenna, not the reaction center, determines the excited-state lifetime - Both the maximum and the nonphotochemically quenched', *Biophysical Journal*, 102(12), pp. 2761–2771. Available at: https://doi.org/10.1016/j.bpj.2012.05.004.

Berry, M.W. *et al.* (2007) 'Algorithms and applications for approximate nonnegative matrix factorization', *Computational Statistics and Data Analysis*, 52(1), pp. 155–173. Available at: https://doi.org/10.1016/j.csda.2006.11.006.

Franssen, W.M.J. *et al.* (2020) 'Full-Harmonics Phasor Analysis: Unravelling Multiexponential Trends in Magnetic Resonance Imaging Data', *The Journal of Physical Chemistry Letters*, 11(21), pp. 9152–9158. Available at: https://doi.org/10.1021/acs.jpclett.0c02319.

Fritzsch, R. *et al.* (2018) 'Rapid Screening of DNA–Ligand Complexes via 2D-IR Spectroscopy and ANOVA–PCA', *Analytical Chemistry*, 90(4), pp. 2732–2740. Available at: https://doi.org/10.1021/acs.analchem.7b04727.

Maćkiewicz, A. and Ratajczak, W. (1993) ‘PRINCIPAL COMPONENTS ANALYSIS (PCA)*’, *Computers & Geosciences*, 19(3), p. 303.

Ramakers, L.A.I. *et al.* (2025) ‘Unravelling the different components of nonphotochemical quenching using a novel analytical pipeline’, *New Phytologist*, 245, pp. 625–636. Available at: https://doi.org/10.1111/nph.20271.
